# Maintenance of active chromatin states by HMGN2 is required for stem cell identity in a pluripotent stem cell model

**DOI:** 10.1186/s13072-019-0320-7

**Published:** 2019-12-12

**Authors:** Sylvia Garza-Manero, Abdulmajeed Abdulghani A. Sindi, Gokula Mohan, Ohoud Rehbini, Valentine H. M. Jeantet, Mariarca Bailo, Faeezah Abdul Latif, Maureen P. West, Ross Gurden, Lauren Finlayson, Silvija Svambaryte, Adam G. West, Katherine L. West

**Affiliations:** 1 Institute of Cancer Sciences, College of Medical Veterinary and Life Sciences, University of Glasgow, University Avenue, Glasgow, G12 8QQ UK; 2 School of Life Sciences, College of Medical Veterinary and Life Sciences, University of Glasgow, University Avenue, Glasgow, G12 8QQ UK; 3 Present Address: Institute of Biological Sciences, Faculty of Science, University of Malaya, Kuala Lumpur, Malaysia; 4 Present Address: Department of Basic Medical Sciences, Faculty of Applied Medical Sciences, Albaha University, Albaha-Alaqiq, Saudi Arabia

**Keywords:** HMGN, Chromatin, Epigenetics, Stem cells, Embryonal carcinoma cells, Differentiation, Neuronal

## Abstract

**Background:**

Members of the HMGN protein family modulate chromatin structure and influence epigenetic modifications. HMGN1 and HMGN2 are highly expressed during early development and in the neural stem/progenitor cells of the developing and adult brain. Here, we investigate whether HMGN proteins contribute to the chromatin plasticity and epigenetic regulation that is essential for maintaining pluripotency in stem cells.

**Results:**

We show that loss of *Hmgn1* or *Hmgn2* in pluripotent embryonal carcinoma cells leads to increased levels of spontaneous neuronal differentiation. This is accompanied by the loss of pluripotency markers *Nanog* and *Ssea1*, and increased expression of the pro-neural transcription factors *Neurog1* and *Ascl1*. Neural stem cells derived from these *Hmgn*-knockout lines also show increased spontaneous neuronal differentiation and *Neurog1* expression. The loss of HMGN2 leads to a global reduction in H3K9 acetylation, and disrupts the profile of H3K4me3, H3K9ac, H3K27ac and H3K122ac at the *Nanog* and *Oct4* loci. At endodermal/mesodermal genes, *Hmgn2*-knockout cells show a switch from a bivalent to a repressive chromatin configuration. However, at neuronal lineage genes whose expression is increased, no epigenetic changes are observed and their bivalent states are retained following the loss of HMGN2.

**Conclusions:**

We conclude that HMGN1 and HMGN2 maintain the identity of pluripotent embryonal carcinoma cells by optimising the pluripotency transcription factor network and protecting the cells from precocious differentiation. Our evidence suggests that HMGN2 regulates active and bivalent genes by promoting an epigenetic landscape of active histone modifications at promoters and enhancers.

## Background

The chromatin landscape of embryonic stem cells has distinct characteristics that are believed to confer increased plasticity and thus contribute to the maintenance of the pluripotent state [1]. Plasticity is achieved through a range of mechanisms, including bivalent epigenetic states [2], more numerous DNaseI-hypersensitive sites [3], increased levels of active transcriptional marks, fewer and less condensed heterochromatin loci, and hyperdynamic binding of major architectural proteins such as the linker histones [4].

HMGN (high mobility group nucleosome binding) family members are chromatin architectural proteins that influence local and global chromatin structure, and have wide ranging effects on gene expression [5]. They are devoid of enzymatic activity, and bind to the nucleosome or core particle of chromatin without specificity for the underlying DNA sequence. The two most abundant and well-studied HMGN isoforms are HMGN1 and HMGN2. The five HMGN family members share a general structure, consisting of a conserved positively charged nucleosome-binding domain (NBD) that specifically targets the nucleosome and constitutes the hallmark of the family, a bipartite nuclear localisation signal, and a variable negatively charged C-terminal or regulatory domain (RD) [6].

HMGN proteins have been shown to modulate chromatin structure in a variety of ways. They can alter the ability of histone acetyl-transferases and kinases to modify core histone N-terminal tails [7–9] and have been shown to inhibit the activity of chromatin remodelling complexes [10]. They also inhibit the interaction of linker histones with chromatin, which can lead to decompaction and reduced EZH2-mediated methylation of histone H3K27 [11, 12]. They have also been shown to increase the recruitment of certain transcription factors to chromatin [13].

Chromatin immunoprecipitation studies from several labs have shown that HMGN proteins do not tend to be highly enriched at individual genes [9, 14–16]. This is consistent with observations that HMGNs do not have any specificity for the underlying DNA sequence, and that their binding to chromatin is transient and dynamic, and with continuous exchange from one nucleosome to another [17]. However, ChIP-seq studies by the Bustin lab using different antibodies have shown that HMGN1 and HMGN2 are enriched at DNase I hypersensitivity sites (DHSs) in several mammalian cell lines [3, 18–20]. DHSs constitute chromatin-accessible domains located at transcriptional regulatory regions, such as promoters and enhancers, and are considered to be a hallmark of genes poised or activated for transcription. Cells lacking both HMGN1 and HMGN2 exhibit a reduction in the number and the intensity of the DHSs in mouse embryonic fibroblasts and mouse embryonic stem cells (ESCs) [19, 20].

The generation of *Hmgn* variant-specific knockout mice in the last decade has provided considerable insights into the relevance of these proteins at the cellular and organismal level. In general, these mice are viable and do not present strong phenotypes. Nevertheless, variant-specific phenotypic alterations have been reported. For example, *Hmgn1*^−/−^ mice exhibit increased tumorigenicity and impaired DNA damage response [21], whereas *Hmgn2*^−/−^ mice show some alterations in energy metabolism [19]. Analyses of transcriptional changes in cells and tissues derived from the genetically modified mice indicate that the absence of one or two *Hmgn* variants does not dramatically modify the pre-existing transcriptional profile [3, 12, 19, 20]. However, there are significant changes in the levels of many mRNA transcripts, which indicate that the general process of transcription is altered, and accounts for the *Hmgn* knockout mouse phenotypes mentioned above. These experiments led to the working hypothesis that the HMGN proteins fine-tune an already established expression profile, and ensure the appropriate cellular response to external and internal cues.

During development, HMGN1 and HMGN2 are highly abundant in embryonic tissues, and then are progressively downregulated as differentiation proceeds, remaining at lower levels in fully differentiated tissues [22]. This decreasing expression pattern has been observed during erythropoiesis, myogenesis, and chondrogenesis, and seems to be essential, since HMGN1 overexpression inhibits normal cellular differentiation [15, 23, 24]. Although fully differentiated cells have lost substantial amounts of HMGN proteins, many tissue-specific stem cells and transient amplifying precursors retain high levels of these proteins [22, 25].

In the adult mouse brain, *Hmgn1* and *Hmgn2* mRNAs are highly expressed in the dentate gyrus of the adult hippocampus [3], which is a well-characterised neurogenic niche where neural stem cells (NSCs) reside and undergo active neurogenesis. A role for HMGN proteins in NSCs is supported by the observation that adult *Hmgn1*^−/−^ mice exhibit a reduction in nestin-positive NSCs in the sub-ventricular zone [3]. Moreover, HMGN proteins are required for proper differentiation of NSCs into astrocytes and oligodendrocytes, since *Hmgn* knockdown interferes with the neuron-to-glia transition, and the lack of both HMGN1 and HMGN2 leads to downregulation of OLIG1 and OLIG2 with defects in oligogenesis [12, 26]. Defects in neural stem cell differentiation could also be a contributing factor to the neurological defects observed in *Hmgn1*^−/−^ and double knockout *Hmgn1*^−*/*−^*Hmgn2*^−/−^ mice [12, 27]. Together these data suggest a role for HMGN proteins in tissue-specific stem cell biology and differentiation, especially among the neural lineage.

In this study, we have investigated the role of HMGN1 and HMGN2 in P19 embryonal carcinoma stem cells. P19 cells are pluripotent embryonal carcinoma cells that maintain their phenotype when cultured in the presence of serum. They were originally derived from post-implantation embryos [28], capturing a later developmental stage than mouse ES cells. P19 cells resemble epiblast stem cells, a primed state of pluripotency which shares several features with human ES cells [29–31]. P19 cells can be easily differentiated down the neuronal or cardiomyocyte lineages, and have been extensively studied over the last three decades. Our data indicate that HMGN proteins play roles in regulating the active epigenetic landscape and gene expression profile of embryonal carcinoma cells, and thus help to maintain self-renewal and prevent inappropriate differentiation of these pluripotent stem cells.

## Results

### Generation of *Hmgn2* and *Hmgn2* knockout cell lines

**Fig. 1:**
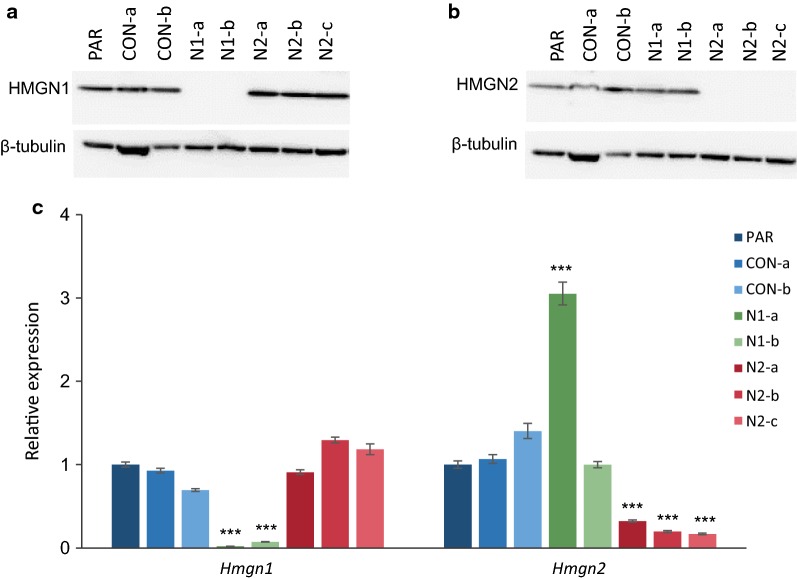
Generation of *Hmgn1* and *Hmgn2* knockout P19 lines. Western blot analysis of **a** HMGN1 and **b** HMGN2 protein levels in clonal P19 lines; β-tubulin is shown as a loading control. **c** *Hmgn1* and *Hmgn2* mRNA expression as determined by qRT-PCR in control and *Hmgn*-knockout cells. The graph represents the average fold change relative to parental P19 cells (*n* = 3–10) (***p < 0.0001)

CRISPR mutagenesis [32] was used to create clonal derivatives of murine P19 embryonal carcinoma cells in which HMGN1 or HMGN2 protein expression was abolished (Additional file 1: Figure S1). Two independent lines show complete loss of HMGN1 expression: N1-a and N1-b (Fig. 1a). Control line CON-b went through the same process but *Hmgn1* was not targeted and no loss of HMGN1 protein is observed. Three independent lines show complete loss of HMGN2 protein expression: N2-a, N2-b and N2-c (Fig. 1b). Control line CON-a was generated at the same time, but has no mutagenesis of the *Hmgn2* alleles.

Immunofluorescence confirmed the loss of HMGN1 and HMGN2 protein in all cells in their respective knockout lines (Additional file 1: Figure S2). Quantitative RT-PCR revealed that *Hmgn1* and *Hmgn2* transcript levels are significantly reduced in the knock-out cell lines, indicative of either a change in splicing preferences and/or a reduction in transcript stability (Fig. 1c). The level of the *Hmgn2* transcript is increased by threefold in the *Hmgn1* mutant line N1a (Fig. 1c), but no corresponding increase in HMGN2 protein is observed (Fig. 1b). No other reciprocal changes in HMGN1 or HMGN2 expression were observed in the other knockout or control lines (Fig. 1a–c).

### *Hmgn* knockout cells have reduced expression of pluripotency markers

P19 cells have lost the capacity to spontaneously differentiate, despite their teratocarcinoma origin, and can be propagated indefinitely in serum-containing medium as mostly pure cultures of undifferentiated cells. Parental P19 cells are morphologically homogeneous and grow as discrete colonies. We observed that the loss of HMGN1 or HMGN2 expression considerably alters the cellular morphology and organisation of P19 cultures (Additional file 1: Figure S3). Fewer discrete colonies are observed, with a high proportion of *Hmgn*-knockout cells growing outside of normal colony boundaries. Many of these cells exhibit an extended cytoplasm and resemble differentiated cells; in particular, some cells have long thin processes that connect with other cells (Additional file 1: Figure S3, red arrows).

**Fig. 2:**
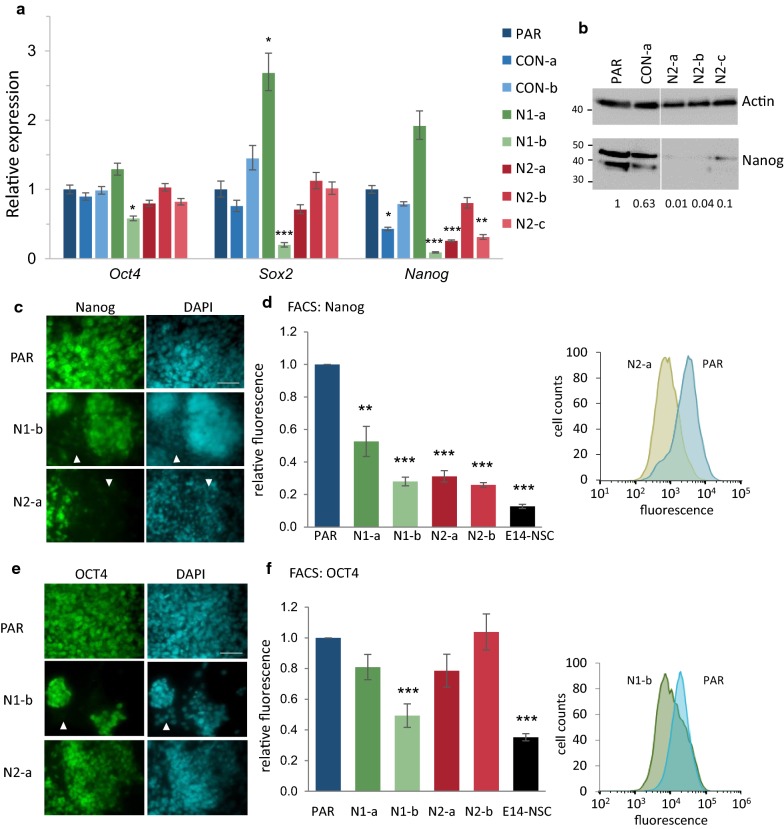
Expression of pluripotency markers in *Hmgn* knockout cells. **a** Relative expression of the pluripotency transcription factors *Oct4*, *Sox2*, and *Nanog* as determined by qRT-PCR. The graphs show the fold change relative to parental P19 cells (*n* = 3–10) (*p < 0.05, **p < 0.01, ***p < 0.001). **b** Western blot analysis of Nanog protein levels in whole cell extracts from parental P19, CON-a, N2-a, N2-b and N2-c cells. For both Actin and Nanog, all samples were run on the same gel and exposed at the same time. Actin is shown as a loading control. The relative ratios of Nanog to Actin, averaged from two replicate blots, are shown below. Immunofluorescence analysis of Nanog (**c**) and OCT4 (**e**) expression. Parental, N1-a and N2-a cells were fixed 24 h after seeding. DAPI was used to stain the nuclei (cyan). Scale bar indicates 50 µm. Nanog levels are heterogeneous, and cells with reduced expression are indicated by arrow heads. Images from all lines are shown in Additional file 1: Figure S4. FACS analysis of Nanog (**d**) and OCT4 (**f**) expression. E14-NSCs are the negative control. The bar graphs represent the relative fluorescence intensity (median) of each cell line, *n* = 3 (*p < 0.05, **p < 0.001, ***p < 0.0001). The cell count plots are representative examples to illustrate the distribution of the data for parental and knockout cells

We hypothesised that these changes in cell morphology in the *Hmgn* knockout populations could reflect an increased propensity to differentiate. In order to investigate this further, the expression of self-renewal markers was assayed by immunofluorescence and qRT-PCR. The core pluripotency transcription factor Nanog is highly expressed in naïve pluripotent cells and is downregulated upon lineage-priming [29, 30]. In the *Hmgn* knockout lines N1-b, N2-a and N2-c, *Nanog* mRNA expression is reduced by 3- to 10-fold relative to parental P19 cells (Fig. 2a). A reduction in Nanog protein in all the *Hmgn2*-knockout lines is also observed by western blotting (Fig. 2b). Immunofluorescence microscopy revealed that Nanog protein expression is reduced in most cells in all the *Hmgn1* and *Hmgn2*-knockout cultures, even in the N1-a and N2-b lines where *Nanog* mRNA levels are not significantly reduced (Fig. 2c and Additional file 1: Figure S4a). The control line CON-a shows a 50% reduction in *Nanog* mRNA (p < 0.05) and a 37% reduction in Nanog protein by western blotting, although no obvious differences are apparent by immunofluorescence. The levels of Nanog protein are heterogeneous, particularly within the knockout lines, which is in agreement with previous reports suggesting that heterogeneity in Nanog production is related to diverse differentiation potentials among a polypopulation of pluripotent cells [33]. A reduction in Nanog protein levels in the knockout lines was confirmed using FACS, which revealed a significant decrease of 50% or more in the median level of Nanog protein production in all four *Hmgn1* and *Hmgn2*-knockout lines tested (Fig. 2d).

Stage-specific embryonic antigen 1 (SSEA1) plays a role in cell adhesion and migration in the pre-implantation embryo and is widely used as a pluripotency marker in mouse ESCs, and is also expressed in embryonal carcinoma cells, including P19 cells [34]. Immunofluorescence microscopy reveals a reduction in SSEA1 in both *Hmgn1* and *Hmgn2*-knockout cells, and quantification by FACS indicates a reduction of 39–65% in SSEA1 protein levels in the knockout lines (Additional file 1: Figure S4c and d).

OCT4 is a transcription factor that is highly expressed in undifferentiated pluripotent stem cells. It has also been shown to be retained in the first stages of ESC differentiation [35]. *Oct4* mRNA levels are similar across all the *Hmgn* knockout lines except N1-b, in which it is decreased by 40% (Fig. 2a). This is consistent with immunofluorescence data (Fig. 2e and Additional file 1: Figure S4b). In the parental cells and in most of the *Hmgn* knockout lines, OCT4 is homogeneously distributed and most of the cells show equivalent fluorescence intensity (Fig. 2e and Additional file 1: Figure S4b). However, some cells growing outwith colony boundaries are completely devoid of OCT4, especially in N1-b cultures (Additional file 1: Figure S4b, arrow heads). FACS analysis shows that median OCT4 expression is unchanged in most of the *Hmgn* knockout lines, but is reduced by 50% in the N1-b line (Fig. 2f).

*Sox2* has a similar expression pattern to *Oct4* in the different pluripotency states and during early ESC differentiation [35], and is also expressed in neural stem cells. *Sox2* mRNA expression is not altered in the *Hmgn2* knockout lines, although its expression is increased in N1-a cells and decreased in N1-b cells (Fig. 2a).

In summary, a reduction in Nanog and SSEA1 protein levels is observed across all the *Hmgn* knockout lines, whereas OCT4 and Sox2 expression is not consistently affected. This data suggests that the maintenance of self-renewal and pluripotency may be compromised in *Hmgn*-knockout cultures.

### *Hmgn* knockout cells show increased spontaneous neuronal differentiation

**Fig. 3:**
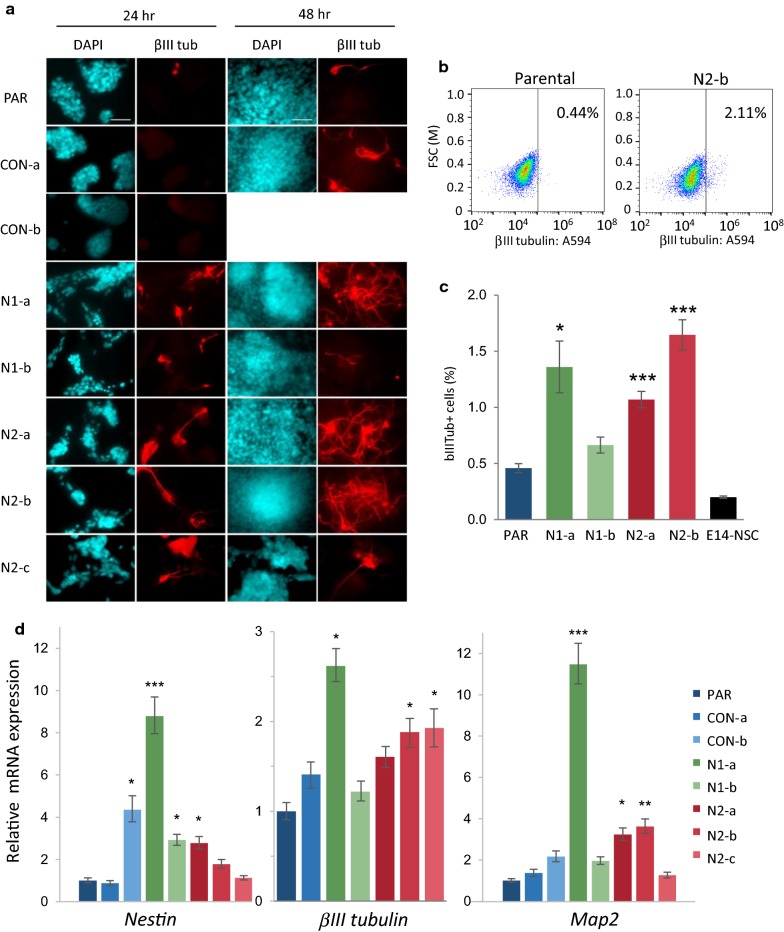
*Hmgn* knockout cells show increased levels of spontaneous neuronal differentiation. **a** Immunofluorescence analysis of the neurofilament protein βIII-tubulin (red). Control and *Hmgn*-knockout cells were fixed 24 h and 48 h after seeding. CON-b cells adhere weakly to slides after 48 h, so no immunofluorescence data are available for this time point. DAPI was used to stain the nuclei (cyan). Scale bar indicates 50 µm. FACS analysis of βIII tubulin expression. **b** Example dot plots show the distribution of 10,000 cells and the gate that delineates βIII tubulin-positive cells. The percentage of βIII tubulin-positive cells is shown. **c** Percentage of βIII tubulin-positive cells in each cell line, as determined by FACS (*n* = 3) (*p < 0.05, **p < 0.001, ***p < 0.0001). E14-NSCs are a negative control. **d** Relative mRNA expression of the neural-specific markers *Nestin*, *βIII*-*tubulin* and *Map2* (no data available for *βIII tubulin* expression in CON-b cells). The graphs show the fold change relative to parental cells (*n* = 3–10) (*p < 0.05, **p < 0.01, ***p < 0.001)

To investigate whether *Hmgn* knockout cells display a loss of pluripotency, the cultures were examined for evidence of spontaneous differentiation down neuronal and endodermal/mesodermal pathways. βIII tubulin immunostaining was performed to detect differentiation down the neuronal lineage (Fig. 3a). Strikingly, 24 h after seeding the cells at low density, all five *Hmgn* knockout lines show increased formation of βIII tubulin-positive cells with extended processes, a phenotype that is typical of immature neuronal cells. This contrasts strongly with the low frequency of βIII tubulin-positive cells that are typically observed in the control cultures (Fig. 3a). At 48 h after seeding, extensive networks of neurites are visible in lines N1-a, N2-a, N2-b and N2-c (Fig. 3a). FACS was used to quantify the percentage of cells that are positive for βIII tubulin, and the data show that N1-a, N2-a and N2-b lines have significant increases in the percentage of βIII tubulin-positive cells (Fig. 3b, c).

Consistent with the observed changes in protein expression and cell morphology, *βIII tubulin* mRNA levels show a small increase in N1-a, N2-b and N2-c cells, and expression of *Map2*, another neuronal cytoskeletal protein, is increased by 3- to 11-fold in several knockout lines (Fig. 3d).

Nestin is an intermediate filament that is as a classical marker of neural stem cells. P19 undifferentiated cells express Nestin at basal levels [36]. *Nestin* mRNA expression is increased by 3- to 9-fold in N1-a, N1-b and N2-a cells, and is also increased by fourfold in one of the control lines, CON-b (Fig. 3d). Immunofluorescence shows that Nestin protein levels are higher in several of the *Hmgn* knockout cultures than in the control lines, particularly in the cells growing in three-dimensional clusters (Additional file 1: Figure S5a, arrows). Quantification by FACS reveals a modest but statistically significant increase of 75% in Nestin expression in two of the knockout lines, N1-a and N2-a (Additional file 1: Figure S5c).

**Fig. 4:**
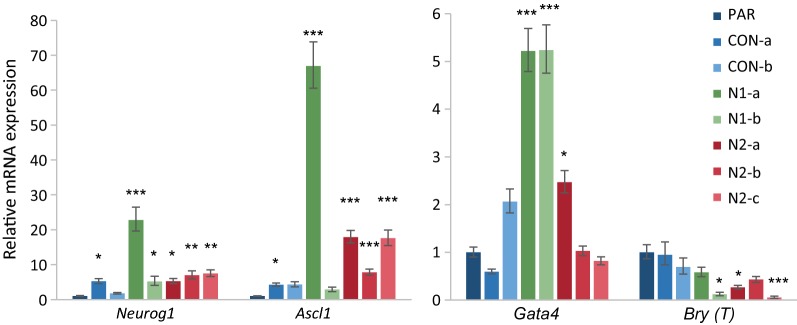
*Hmgn* knockout cells have increased expression of pro-neural transcription factors. Relative mRNA expression of the proneural lineage-specific transcription factors *Neurog1 and Ascl1*, and the endodermal/mesodermal markers *Gata4* and *Bry* (*T*). The graphs show the fold change relative to parental cells (*n* = 3–10) (*p < 0.05, **p < 0.01, ***p < 0.001)

βIII tubulin, Nestin and MAP2 are structural proteins whose expression indicates differentiation down the neural lineage. To investigate the regulation of this process, expression of the pro-neural transcription factors *Neurog1* and *Ascl1* [37] were studied. Consistent with the increased spontaneous neuronal differentiation, *Neurog1* and *Ascl1* are upregulated in most of the *Hmgn*-knockout lines when compared with parental cells (Fig. 4). *Neurog1* transcription is increased by 5- to 25-fold in *Hmgn1*- and *Hmgn2*-knockout lines, and *Ascl1* is increased by 8- to 18-fold in *Hmgn2*-knockout cells and 67-fold in N1-a cells when compared with the parental cells (Fig. 4). We propose that increased expression of *Neurog1* and *Ascl1* drives the spontaneous differentiation of βIII tubulin-positive neuronal cells in *Hmgn* knockout cultures.

P19 cells can be differentiated down the mesodermal lineage into cardiomyocytes in the presence of DMSO or other inducers [38]. To test whether *Hmgn*-knockout cells spontaneously differentiate down this lineage, *Gata4* and *Brachyury* (*Bry* or *T*) expression was analysed. *Gata4* and *Brachyury* are typically regarded as mesodermal and/or endodermal markers. In differentiating P19 cells, *Brachyury* is an early marker of mesodermal differentiation, whereas *Gata4* is expressed slightly later, in cardiomyoblasts, where it regulates cardiomyocyte differentiation [39]. We find that *Gata4* transcript levels are increased by around fivefold in the *Hmgn1* knockout lines N1-a and N1-b, but are only increased by 2.5-fold in one of the *Hmgn2* knockout lines, N2-a (Fig. 4). Immunofluorescence reveals very few GATA4-positive cells across all the control and *Hmgn* knockout cell lines, with slightly more observed in line N1-b (Additional file 1: Figure S5b, arrows). Quantification by FACS shows that the median GATA4 expression is not significantly increased in the *Hmgn* knockout lines (Additional file 1: Figure S5d). Conversely, *Brachyury* transcript levels are decreased in three of the *Hmgn* knockout cell lines (N1-b, N2-a and N2-c) (Fig. 4). Brachyury protein was undetectable by immunofluorescence. Although the increase in *Gata4* mRNA expression might indicate initiation of cardiogenesis, this is not consistent with the reduction in *Brachyury* expression. These data are more consistent with some loss of control of gene regulation in the *Hmgn1* knockout cells.

The timing of differentiation is regulated by transcriptional switches that are driven by intrinsic or extrinsic signals. In order to test whether FGF, WNT or Notch signalling is altered in *Hmgn2* knockout cells, we analysed the expression of a range of genes in the presence and absence of signalling pathway inhibitors (Additional file 1: Figure S6). The data do not support a role for these pathways in promoting differentiation after the loss of *Hmgn2*, so we propose that the initiation of differentiation in the *Hmgn2* knockout cells follows an intrinsic and cell-autonomous programme.

In order to investigate whether *Hmgn* knockout cells show an increased propensity to differentiate into neurons following extrinsic induction stimuli, a well-established P19 neuronal induction protocol that utilises retinoic acid and FGF8 was followed [40] (Additional file 1: Figures S7 and S8). No significant differences in the induction of *Neurog1*, *Ascl1* or *Nestin* are observed between control and knockout cells during differentiation, nor are there differences in the repression of *Nanog* (Additional file 1: Figures S7 and S8). Both control and knockout cells form complex networks of neurites by day 4, and immunofluorescence for βIII tubulin did not reveal any significant differences in the level of marker expression or cell morphology (Additional file 1: Figure S7b). These results indicate that most of the *Hmgn*-knockout cells respond normally to neuronal induction cues, and are capable of generating neuronal cells with similar timing and efficiency to the parental and control lines.

Taken together, these analyses show that the loss of either *Hmgn1* or *Hmgn2* disrupts the gene expression profile of pluripotent embryonal carcinoma cells, compromising the fidelity of self-renewal and leading to the spontaneous initiation of differentiation programmes that are mainly, but not exclusively, towards neural lineages.

### Loss of *Hmgn1* or *Hmgn2* affects the profile of active histone modifications

**Fig. 5:**
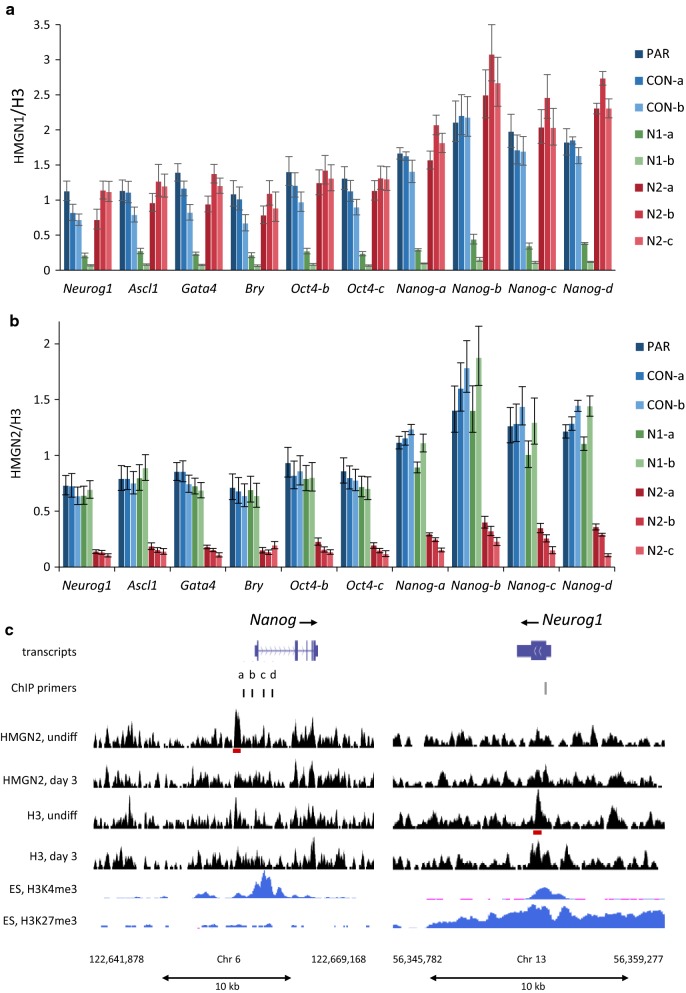
HMGN1 and HMGN2 are not highly enriched at active gene promoters. ChIP-PCR assays in control and knockout cell lines. Enrichment of HMGN1 (**a**) or HMGN2 (**b**) at each primer set was normalised to the average H3 signal from all primer sets. The graphs present the mean and sd of technical qPCR triplicates from the same IP reaction. Data are representative of 2–3 independent biological replicates. **c** ChIP-seq for HMGN2 and H3 was performed in undifferentiated and day 3 neuronal induced P19 cells. Reads were aligned to the mm9 mouse genome and regions surrounding the *Nanog* and *Neurog1* loci are shown. Peaks revealed by MACS peak calling software for HMGN2 and H3 are shown as red blocks below the relevant signal track. Positions of the primer sets used for ChIP are indicated. Data for H3K4me3 and H3K27me3 in mouse ES Bruce4 cells was obtained from the UCSC genome browser (http://genome.ucsc.edu; accessions wgEncodeEM001682 and wgEncodeEM002709) [50, 51]. *Y*-axis maxima are 0.62 for all H3 and HMGN2 tracks, 21 for H3K4me3 and 3.5 for H3K27me3. Similar data for the regions surrounding the *Oct4* and *Ascl1* loci are shown in Additional file 1: Figure S9

We hypothesised that HMGN proteins regulate the expression of *Nanog*, *Neurog1*, *Ascl1*, *Gata4* and *Brachyury* by modulating their epigenetic profiles. Examination of publicly available ChIP-seq data from mouse ES cells shows that the highly expressed *Nanog* and *Oct4* genes have high levels of the active mark H3K4me3 in the vicinity of their transcription start sites (TSS), and negligible levels of the repressive mark H3K27me3 (Fig. 5c, Additional file 1: Figure S9, and data not shown). Notably, *Neurog1*, *Ascl1*, *Gata4* and *Brachyury* have moderate levels of H3K4me3, but also have substantial levels of H3K27me3. This “bivalent” pattern is typical of poised stem cell genes with low levels of expression, which can be upregulated or silenced as the stem cells differentiate [2].

To investigate whether HMGN1 or HMGN2 bind preferentially to the promoters of these genes, chromatin immunoprecipitation followed by qPCR was performed. Binding at the *Oct4* and *Nanog* gene loci was assayed using several primer sets that encompass 2–3 kb surrounding the TSS. Single primer sets for *Neurog1*, *Ascl1*, and *Bry* target the peak of H3K4me3 nearest the TSS of each gene (Fig. 5c and Additional file 1: Figure S9).

The ChIP data shows that HMGN1 and HMGN2 are bound at all the active and poised genes tested. Occupancy varies by less than twofold between different genes, with around 50% higher occupancy at the *Nanog* gene locus (*p* < 0.001) (Fig. 5a, b). In the *Hmgn1* knockout lines, HMGN1 signals are reduced by 80% and 95% in the N1-a and N1-b lines, respectively. Conversely, HMGN2 signals are reduced by 80% on average in the *Hmgn2* knockout lines, N2-a, N2-b and N2-c. These data confirm the specificity of the HMGN1 and HMGN2 ChIP assays, and show that these proteins occupy the regulatory regions of both active pluripotency genes and poised/bivalent lineage-specific genes in undifferentiated P19 cells.

In order to investigate whether HMGN2 binding is particularly enriched at these TSS regions compared to other regions of the genome, ChIP-seq was performed in both undifferentiated P19 cells and in P19 cells after 3 days of induced neuronal differentiation (Fig. 5c and Additional file 1: Figure S9). The active marks H3K27ac and H3K4me1 were also mapped in order to validate the ChIP assay and illustrate the chromatin landscape of undifferentiated and day 3 neuronal P19 populations (Additional file 1: Figure S9). For example, H3K27ac is enriched upstream of the *Oct4* gene in undifferentiated cells, but this is lost in the day 3 neuronal cells when *Oct4* is repressed. Conversely, H3K27ac is absent from the *Ascl1* locus in undifferentiated cells, but increased in the day 3 neuronal cells when the gene is expressed. The H3K27ac and H3K4me1 profiles for undifferentiated P19 cells correspond closely to publicly available datasets for undifferentiated mouse ES cells (Additional file 1: Figure S9).

The HMGN2 ChIP-seq binding profiles indicate that HMGN2 is not more highly enriched at active gene promoters, either in undifferentiated or day 3 neuronal cells, but instead is found at similar levels throughout the genome (Fig. 5c and Additional file 1: Figure S9). In general, the profile of HMGN2 binding tends to follow that of H3 (Additional file 1: Figure S10). Peak calling software identified 1862 HMGN2 peaks in undifferentiated cells. For example, there is a HMGN2 peak in the *Nanog* promoter in undifferentiated cells (Fig. 5c). However, only 8% of these HMGN2 peaks overlap with TSSs or putative enhancers (Additional file 1: Figure S10).

**Fig. 6:**
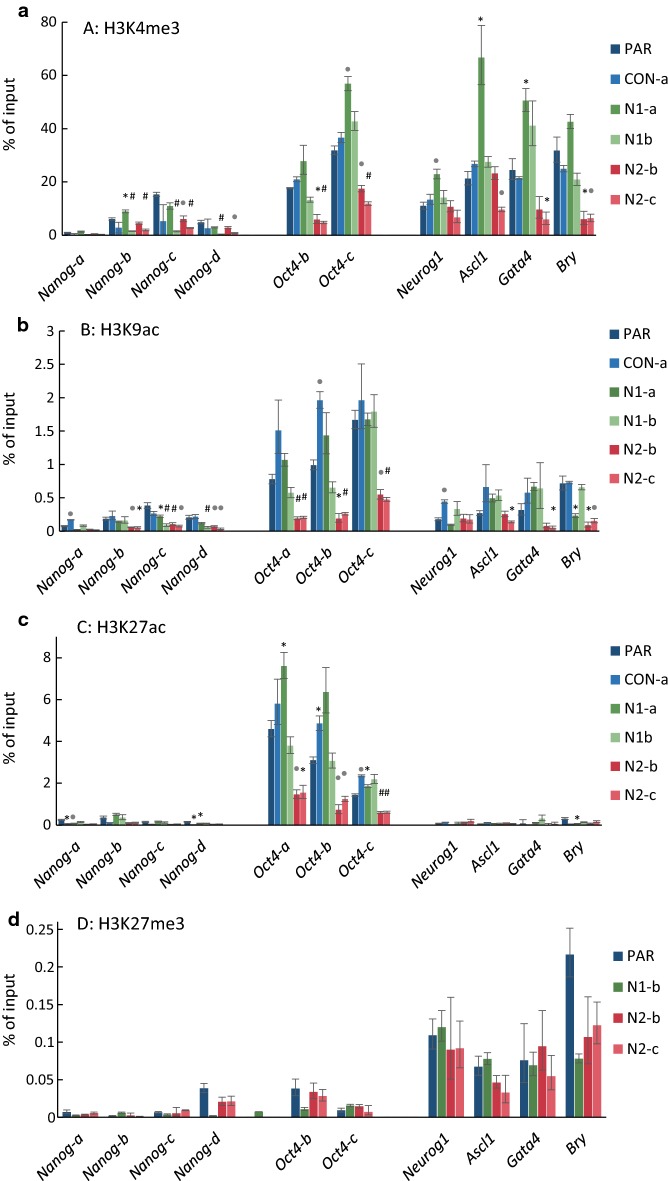
*Hmgn2*-knockout cells show a reduction in active histone H3 modifications at bivalent and active gene loci. ChIP-PCR assays in control and knockout cell lines for H3K4me3 (**a**), H3K9ac (**b**), H3K27ac (**c**) and H3K27me3 (**d**). Data show the enrichment of each modification as a percentage of input. Statistical significance between parental and experimental lines was determined using the Student’s T test, with Bonferroni correction for multiple testing (*p < 0.005, .*p* < 0.001, ^#^*p* < 0.0001). Data for H3K122ac, H3 and H1 are shown in Additional file 1: Figure S11

In order to investigate whether loss of HMGN1 or HMGN2 affects histone modifications at gene promoters, ChIP-PCR was performed in undifferentiated control and knockout cells (Fig. 6 and Additional file 1: Figure S11). At the *Nanog* gene, knockout of *Hmgn2* lead to a loss of the active marks H3K4me3 and H3K9ac, consistent with the reduction in *Nanog* expression in these cells (Fig. 2). Active marks were also reduced in N1-b cells, which have reduced *Nanog* mRNA expression, but not in N1-a cells, where *Nanog* mRNA expression is not reduced.

Unlike the *Nanog* gene locus, the *Oct4* promoter is enriched in the enhancer marks H3K27ac and H3K122ac, as well as in H3K4me3 and H3K9ac. Strikingly, all four of these active marks are reduced in both the *Hmgn2* knockout lines tested (Fig. 6 and Additional file 1: Figure S11). However, they are unchanged or in some cases increased in the *Hmgn1* knockout lines. This is inconsistent with the expression data, where Oct4 expression is unchanged in *Hmgn2* knockouts, but reduced by 40% in N1-b cells.

Reductions in the active marks H3K4me3 and H3K9ac are observed at several of the bivalent gene promoters in *Hmgn2*-knockout lines, indicating a switch from the bivalent state to a more repressive chromatin configuration. These reductions do not always correlate with changes in gene expression (Fig. 6a, b). For example, H3K4me3 and H3K9ac are reduced at *Ascl1* in N2-c cells, even though *Ascl1* expression is increased by 17-fold in these cells (Fig. 4). Furthermore, H3K4me3 and H3K9ac are reduced at the *Brachyury* locus in both N2-b and N2-c cells, but expression of *Brachyury* is only significantly decreased in the N2-c line.

In contrast, the loss of *Hmgn1* does not lead to a reduction in active marks at the bivalent genes. Indeed, in N1-a cells, H3K4me3 levels are increased at *Neurog1*, *Ascl1* and *Gata4*, correlating with the increase in expression of these three genes in N1-a cells (Fig. 4).

Enrichment of the repressive mark H3K27me3 at bivalent gene promoters was not altered in any of the lines tested (Fig. 6d). Nor was there any increase in the level of histone H1 binding (Additional file 1: Figure S11c).

**Fig. 7:**
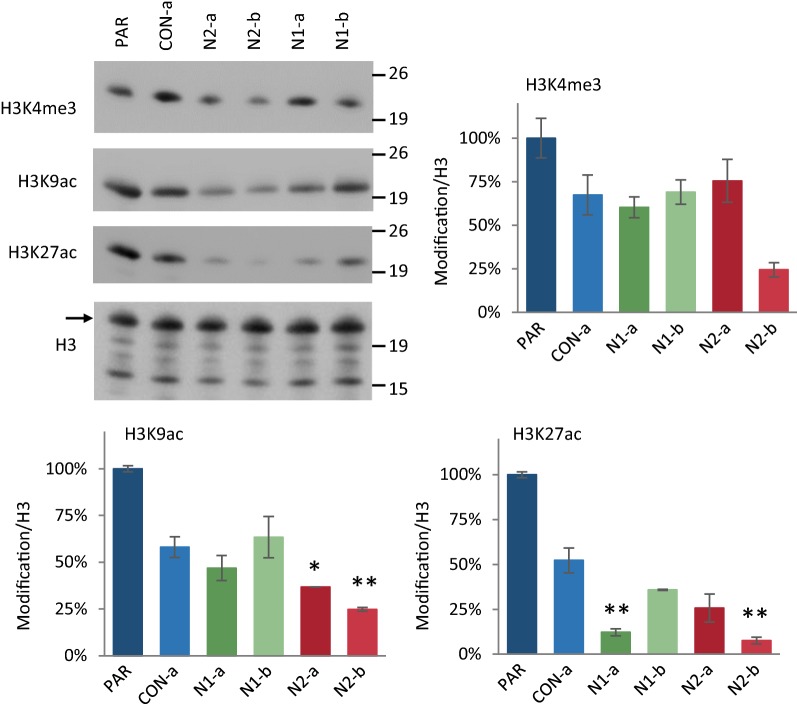
Global reduction of histone acetylation in *Hmgn*-knockout cells. Western blotting for the detection of H3K4me3, H3K9ac and H3K27ac. Acid histone extracts were prepared in duplicate from two different cell passages of each cell line and separated by SDS-PAGE. Western blotting for each histone modification and sample set was performed twice or three times. Representative images are displayed (left). The H3 protein is indicated by an arrow, and additional bands on this blot are the results of non-specific antibody interactions. The intensity for each modification was normalised to that of H3, and the average and s.e.m. is plotted relative to that in parental cells (*p < 0.05, **p < 0.01)

In order to extend the observations made at individual genomic locations, the global levels of several active histone modifications were investigated in *Hmgn1* and *Hmgn2* knockout cells by western blotting (Fig. 7). Consistent with the ChIP data, both the *Hmgn2* knockout lines tested show a reduction of over 60% in global H3K9ac levels. The largest reduction was observed with H3K27ac, which is reduced by over 74% in the N1-a, N2-a and N2-b knockout cells (Fig. 7).

In summary, it can be concluded that the loss HMGN2 leads to a reduction in active promoter and enhancer marks at most genes tested, irrespective of any changes in expression of these genes, and a global reduction in H3K9 acetylation. The effect of HMGN1 loss is less consistent between different knockout lines, and changes in active histone modifications are more likely to correlate with changes in gene expression.

### Neural stem cells lacking HMGN1 or HMGN2 show loss of NSC self-renewal

**Fig. 8:**
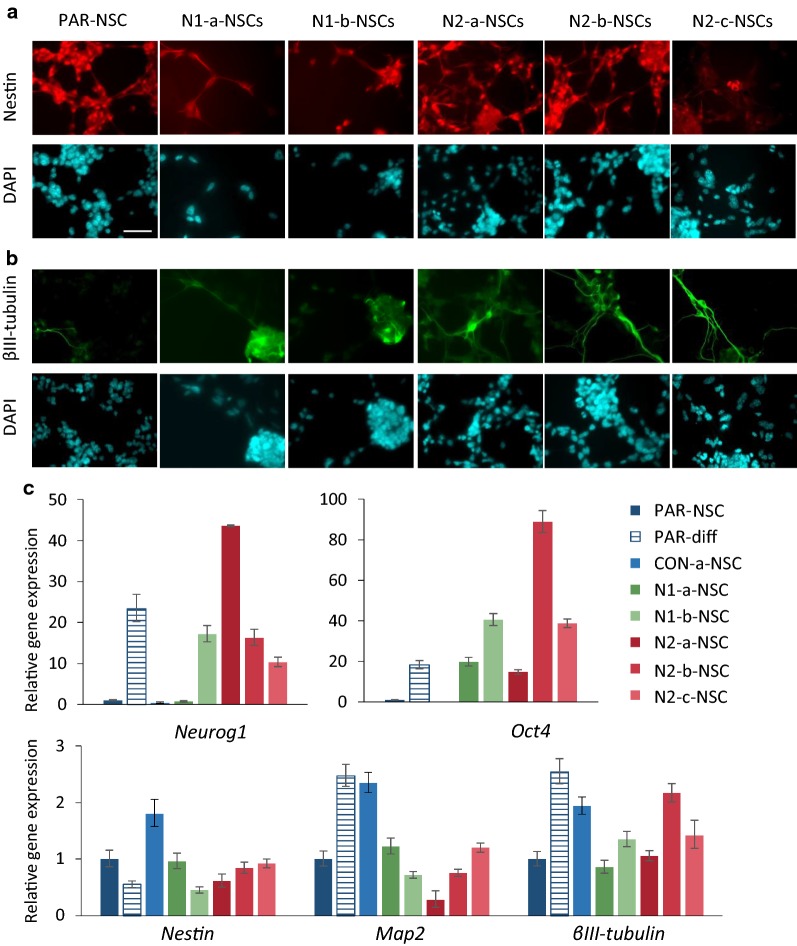
Neural stem cells derived from cells lacking *Hmgn1* or *Hmgn2* show loss of NSC identity. Immunostaining for **a** nestin and **b** βIII tubulin in NSCs derived from parental P19 cells, *Hmgn1* and *Hmgn2* knockout lines. Scale bar indicates 50 µm. **c** Relative mRNA expression in NSCs derived from parental P19 cells, CON-a cells, and *Hmgn1* and *Hmgn2* knockout lines. Expression in parental NSCs that were induced to differentiate down the neuronal lineage by removal of growth factors is also shown. Expression is normalised to that of *Gpi1*, and is plotted relative to that in parental NSCs. Error bars represent the sd from technical qRT-PCR triplicates. Data are representative of two independent NSC derivation experiments

The data presented above shows that the loss of HMGN proteins compromises the self renewal and pluripotency of embryonal carcinoma pluripotent stem cells. To investigate whether the properties of other types of stem cell are also compromised, we derived neural stem-like cells (NSCs) from the control and *Hmgn*-knockout P19 cultures [41]. NSCs derived from parental P19 cells can be maintained for many passages, express high levels of Nestin and have a bipolar morphology that is typical of NSCs (Fig. 8a, PAR-NSC). Parental P19 NSCs can also be differentiated into mixed cultures of neurons and glia upon removal of the growth factors EGF and FGF (e.g. Fig. 8c, PAR-diff columns).

Immunostaining of *Hmgn*-knockout NSC cultures does not reveal any substantial differences in Nestin expression compared to the parental NSC cultures (Fig. 8a). However, higher numbers of βIII-tubulin-positive cells with extended processes are apparent in both *Hmgn1*- and *Hmgn2*-knockout cultures (Fig. 8b). At the mRNA level, neither *Nestin* nor *βIII*-*tubulin* expression is consistently altered in the *Hmgn*-knockout cells, which may indicate that βIII-tubulin protein production is regulated post-transcriptionally in these cells (Fig. 8c).

Consistent with the increased numbers of neuronal cells detected by immunofluorescence, mRNA expression of the pro-neural transcription factor *Neurog1* is increased by 10- to 44-fold in NSCs from N1-1, N2-a, N2-b and N2-c cells compared to the parental NSCs (Fig. 8c). This increase is comparable to the 23-fold increase observed when parental NSCs differentiate into neuronal cultures (PAR-NSC versus PAR-diff, Fig. 8c). Similarly, *Oct4* expression is increased by 15- to 89-fold in all the *Hmgn1*- and *Hmgn2*-knockout NSCs compared to the control NSCs. This is comparable to the 18-fold increase in *Oct4* expression that is observed when parental NSCs differentiate into neuronal cultures (Fig. 8c). However, expression of neuronal marker *Map2* is not consistently altered in *Hmgn* knockout cultures compared to the parental and control NSCs.

These data indicate that HMGN1 and HMGN2 play roles in maintaining the self-renewal of NSCs derived from P19 cells. The loss of either variant leads to spontaneous neuronal differentiation, with increased *Neurog1* expression and production of βIII-tubulin-positive cells.

## Discussion

### HMGN proteins regulate the self-renewal of pluripotent and multipotent embryonal carcinoma cells

The results of the present work demonstrate that the loss of HMGN1 or HMGN2 compromises the ability of embryonal carcinoma cells, and the neural stem cells derived from them, to self-renew accurately and maintain pluri/multipotency. As a consequence, a proportion of the cells exit from the stem cell state and initiate differentiation programmes. A role for HMGN proteins in regulating stem cell identity is consistent with observations that they are highly expressed during development and in differentiating cells, but are downregulated in mature tissues [15, 22]. In particular, high HMGN expression has been observed in the neural stem/progenitor cells of the sub-ventricular zone of the mouse brain, and the transit-amplifying progenitor cells of hair follicle [3, 22, 25, 26]. Furthermore, downregulation of HMGN expression is important for the differentiation of limb bud mesenchymal cells into chondrocytes in vitro [15, 24].

Here, we show that a high proportion of *Hmgn1* and *Hmgn2*-knockout cells have reduced expression of the pluripotency markers SSEA1 and Nanog. Spontaneous differentiation of *Hmgn*-knockout cells down the neural lineage is evidenced by βIII-tubulin immunostaining. Consistent with this, gene expression analyses show increased *Neurog1* and *Ascl1* expression in *Hmgn1* and *Hmgn2* knockout lines. It is important to note that there is some phenotypic variation between clonal lines, indicating that other factors can also influence the rate of spontaneous differentiation of these embryonal carcinoma cells. The predominance of neuronal cells over other lineages is consistent with the default model of neural differentiation, which proposes that pluripotent embryonic stem cells commit to neural fates unless instructed otherwise [42]. For example, ESCs start losing their identity upon withdrawal of the factors that maintain them in a pluripotent state, and they differentiate mostly into neural lineages [41]. NEUROG1 and ASCL1 are powerful pro-neural regulators whose overexpression can drive the neuronal differentiation of P19 cells and ES cells [43, 44]. We propose that these two transcription factors are key contributors to the spontaneous neuronal differentiation observed in the *Hmgn*-knockout cells.

Our observations appear to contrast with previous work performed in mouse ES cells derived from *Hmgn1*^−/−^ and *Hmgn1*^−*/*−^*n2*^−*/*−^ mice, where ES cell pluripotency and self-renewal did not appear to be altered [3, 12]. It is unclear whether the differences are related to the transformed nature of the EC cells, or due to the growth media used, which may permit or restrict spontaneous differentiation depending on whether cells are cultured in the presence of serum or 2i inhibitors. It is also worth considering the different developmental stages from which ESCs and P19 cells were derived. ESCs capture the naïve state of pluripotency from pre-implantation embryos, whereas epiblast stem cells represent a pluripotency state primed for differentiation [29–31]. If this is valid for transformed cells, P19 cells that were derived from post-implantation embryos can immediately commit to differentiation, whereas ESCs first need to exit the naïve pluripotency stage so that they may respond to inductive cues [30]. Therefore, HMGN proteins may not be relevant in the preservation of the naïve pluripotency; rather, these chromatin architectural proteins may be important for keeping the primed cells unresponsive to misplaced inductive differentiation cues.

Analysis of derived neural stem/progenitor cells revealed that the loss of control of self-renewal is not restricted to pluripotent P19 cells, but is also apparent in multipotent P19-NSCs lacking *Hmgn1* or *Hmgn2*. It has been previously demonstrated that *Hmgn1*^−/−^ mice have fewer Nestin-positive cells in the sub-ventricular region of the brain [3], which is consistent with the hypothesis that self-renewal is compromised in neural stem/progenitor cells lacking HMGN1 or HMGN2. Our results complement a previous study that demonstrated a role for HMGN proteins in regulating the differentiation of neural stem/progenitor cells (NPCs) [26]. Overexpression of HMGN proteins in NPCs, both in vivo and in vitro, was shown to promote astrocyte differentiation and inhibit neurogenesis, whereas knocking down *Hmgn* expression had the converse effect [26].

### Hmgn proteins modulate the epigenetic landscape of EC cells

The binding of HMGN proteins to nucleosomes is known to be transient and dynamic [17]. Here, ChIP-seq shows HMGN2 is bound throughout the non-repetitive genome, and with no highly enriched peaks at transcription start sites or enhancers in pluripotent P19 cells and early neuronal cultures. Its binding profile is broadly similar to that of the core histone H3. Previous studies have indicated that HMGN proteins are enriched at DHSs in some cell types but not in others, and we suggest that the use of different antibodies may account for the differences in binding profiles [3, 9, 12, 15, 16, 18–20]. HMGN post-translational modifications, protein conformation or binding partners could differentially influence the affinities of the antibodies. More detailed ChIP-qPCR analysis in P19 cells confirmed that both HMGN1 and HMGN2 are bound at the promoter regions of pluripotency-associated genes, bivalent endodermal/mesodermal lineage genes, and bivalent pro-neural genes.

Chromatin immunoprecipitation assays presented here show that the loss of HMGN2 significantly impacts the epigenetic landscape of P19 cells, leading to reductions in some histone modifications associated with promoter and enhancer activity. Epigenetic marks at both highly expressed, pluripotency-associated factors and at bivalent, lineage-specific genes are reduced, although the extent of the changes and the impact on gene expression varies. There is not a consistent relationship between the changes in expression and the changes in histone modifications at individual genes, so we conclude that the reduction in active marks in *Hmgn2*-knockout cells is not simply a consequence of reduced transcription. The effect of the *Hmgn1*-knockout on epigenetic changes is less consistent between lines, and there is a stronger correlation between changes in active histone modifications and changes in gene expression. Global reductions in the levels of H3K9 and H3K27 acetylation indicate that epigenetic changes are likely to be found at other genes; however, genome-wide studies would be required to investigate this further.

Deng et al. have previously shown that the double knockout of both HMGN1 and HMGN2 leads to increased levels of the repressive mark H3K27me3 around some gene loci in mouse ES cells [12]. This is mediated by increased H1 binding in the absence of HMGN proteins, leading to increased recruitment of the H3K27 methyltransferase, EZH2. However, in P19 cells lacking only HMGN2, we do not observe increased H3K27me3 levels or H1 binding, so it is possible that the remaining HMGN1 is sufficient to prevent increased H1 recruitment.

Several previous studies have shown that HMGN proteins directly influence the levels of histone H3 modifications. Specifically, HMGN1, HMGN2 and HMGN3a can stimulate the ability of PCAF to acetylate nucleosomal H3K14, with HMGN2 showing the greatest effect [6, 8, 9]. This activity is dependent on the C-terminal regulatory domain, and kinetic and structural data reveal that interaction of the HMGN protein with the H3 N-terminal tail may make it a better substrate for histone acetyl-transferase activity [8, 11]. Conversely, HMGN proteins have been shown to inhibit phosphorylation of H3 at serine 10 and serine 28 [6, 7]. Further investigation is needed to determine whether HMGN proteins can also influence the deposition or removal of H3K4 methylation directly.

We conclude that the loss of HMGN2 leads to a widespread changes in the levels of active modifications at histone H3. It is not possible to distinguish between a direct effect of HMGN proteins on the chromatin structure of these genes, and an indirect effect mediated through the expression of other chromatin modifiers; indeed, it is likely that several mechanisms are involved. It is also worth noting that the presence of sequence-specific transcription activators or repressors may override the effect of changes in histone modifications on the expression of individual genes. Further work is required to determine whether other features such as nucleosome positioning, histone variant deposition or transcription factor recruitment are altered in the *Hmgn* knockout cells and could affect the transcriptional profile and cellular phenotype [10, 13, 14].

## Conclusion

We conclude that HMGN1 and HMGN2 maintain P19 cell identity by optimising the pluripotency transcription factor network and protecting the cells from precocious differentiation. We propose that HMGN2 accomplishes this promoting an epigenetic landscape of active histone modifications at promoters and enhancers. Interestingly, a recent paper from Bustin and co-workers shows that the binding of HMGN proteins to cell type-specific enhancers stabilises cell identity [45]. The authors observed that loss of HMGNs enhances the rate of reprogramming of mouse embryonic fibroblasts into induced pluripotent stem cells following the ectopic expression of POU5F1, SOX2, KLF4, and MYC [45]. Beyond the relevance for cellular reprogramming, the paper demonstrates that HMGNs are required to maintain a chromatin organisation that responds appropriately to biological cues [45], which is consistent with our conclusions from the present work. The emerging picture is that HMGN proteins increase the threshold required for cells to respond to signalling events that induce different cellular fates. They do this by working together to promote an active histone modification landscape and open chromatin conformation at regulatory regions of cell type-specific genes, thus stabilising the transcriptional networks.

## Materials and methods

### Cell culture

Mouse embryonal carcinoma cell line, P19, was maintained in advanced DMEM/F12 containing 10% NBCS and 1X GlutaMAX. Adherent neural induction was performed using the method of Nakayama et al. [40], which involved seeding cells onto laminin-coated culture dishes in neural induction media: DMEM/F12 containing sodium pyruvate and NEAA supplemented with 1 X N2 supplement, 200 mM l-glutamine, 500 nM retinoic acid, 10 ng/ml FGF8 and 10 µM DAPT.

To derive neural stem cells, the adherent neural induction protocol was initiated, and after 3 days, cells were switched to gelatine-coated flasks in N2B27 medium containing 10 ng/ml FGF2 and 10 ng/ml EGF [41]. Cells were passaged eight times before being assayed for NSC characteristics. To differentiate the parental NSCs, cells were plated on laminin-coated dishes in N2B27 without FGF and EGF, and assayed after 4 days.

Neural stem cells derived from mouse ES cells (E14-NSCs) were used as controls for FACS, and were derived from E14 cells cultured in 2i media as previously described [41]. For differentiation into glia cells, E14-NSCs were seeded onto laminin coated wells in N2B27 media supplemented with 1% new born calf serum [46]. Medium was replaced every 2 days and harvested for analysis 1 week after growth factor deprivation.

### CRISPR mutagenesis

A dual CRISPR nickase strategy [32] was used to generate frameshift mutations in exon I of *Hmgn2* (Additional file 1: Figure S1). A plasmid expressing the nickase mutant Cas9D10A-T2A-EGFP was transfected alongside plasmids expressing two gRNAs targeting exon I and intron I of *Hmgn2* (gRNA sequences listed in Additional file 2: Table S1). Clonal lines were screened for HMGN2 protein expression by immunofluorescence and western blotting. PCR followed by TA cloning and sequencing was used to confirm mutagenesis of both alleles in each clonal line. Lines N2-a, N2-b and N2-c carry different genetic changes, and so are independent of each other. Control line CON-a was generated at the same time, but has no mutagenesis of the *Hmgn2* alleles. To generate frameshift mutations in exon I of *Hmgn1*, a plasmid expressing wild-type Cas9-T2A-EGFP was co-transfected alongside two gRNA expression plasmids, one expressing targeting *Hmgn1* and the other targeting *Hypoxanthine Phosphoribosyltransferase 1 (Hprt)*. Selection with 10 μg/ml 6-thioguanine was used to isolate clonal lines with inactivating *Hprt* mutations, in order to enrich for cells in which active CRISPR-mediated mutagenesis had occurred. Lines lacking *Hmgn*1 expression were identified as above. Lines N1-a and N1-b were independently derived from separate transfections. Control line CON-b went through the same process, but *Hmgn1* was not targeted and no loss of HMGN1 protein is observed.

### Gene expression analysis

RNA was purified using the Qiagen RNeasy kit with DNase treatment, and quality and quantity were checked by UV spectroscopy (Nanodrop). cDNA was synthesised from 300 ng RNA using Superscript III (Life Technologies) and oligo dT primers, and 5% was used in qRT-PCR reactions with SYBR green Fast start universal master mix (Roche) on a Stratagene MxPro machine. Reaction conditions were 10 min 95 °C, then 40 cycles of 30 s at 95 °C, 1 min at 60 °C. All qRT-PCR and qPCR primer sets were designed to target non-repetitive regions and were checked for the formation of a single product, specificity, and lack of primer dimer formation. No-RT controls were performed for each RNA prep. Amplification efficiency was between 1.92 and 2 in all cases.

The housekeeping gene *Gpi1* was used as the normaliser in ΔΔCt calculations. qRT-PCR primers for *Hmgn1* span the exon V–VI junction, and primers for *Hmgn2* span the exon IV–V junction. Primer sequences and final reaction concentrations are listed in Additional file 2.

### Western blotting

The HMGN1 and HMGN2 antibodies were raised in rabbit by Eurogentec against the C-terminal peptides NQSPASEEEKEAKSD and KTDQAQKAEGAGDAK, respectively, and affinity purified [9]. Western blotting of purified recombinant proteins indicates that these antibodies are specific to the HMGN isoform they were raised against, and this was confirmed using extracts from cells over- or under-expressing specific isoforms (not shown). Acid histone extracts were prepared by incubating cells in triton extraction solution (4.5 μg/ml triton X-100, 4 mM sodium butyrate and protease inhibitors in PBS) for 10 min at 4 °C, washing the cells in the same buffer, then incubating them in 0.2 M HCl for 3 h at 4 °C. Following centrifugation at 300 RFC at 4 °C for 10 min, the supernatant contains the extracted histones. Acid histone extracts were run on 15% SDS-PAGE gels, blotted onto PVDF and membranes probed with the relevant antibodies and HRP-conjugated secondary antibodies. The antigen–antibody reaction was detected with chemiluminescence using a CCD camera imaging system (LAS 3000 Fujifilm) and quantification was performed in Image J. No additional bands were observed other than those shown in the figures. The concentrations of all antibodies used in each experiment are provided in Additional file 2.

### Immunofluorescence

Cells were fixed with 4% PFA for 30 min, permeabilised for 10 min with 0.1% PBS-triton X-100, washed with 20 mM PBS-glycine and blocked with 5% horse serum in 0.5% PBS-triton X-100. Primary antibodies from Abcam were against OCT4 (Ab19857), Nanog (Ab80892), SSEA1 (Ab16285), GATA4 (Ab84593), Nestin (Ab6142), βIII tubulin (Ab18207). Secondary antibodies were from Thermo Fisher Scientific (A21206, A11037, A11020). Images shown are representative of the whole slide.

### FACS

Harvested cells were stained with zombie yellow (for dead cells) then fixed with 4% paraformaldehyde. For SSEA1 detection, cells were incubated with primary antibody in 2% BSA–PBS. For other antibodies, cells were permeabilised and blocked for 15 min in FACS buffer (5% horse serum and 0.5% tween 20 in PBS) before being incubated with primary antibody in FACS buffer. Primary and secondary antibodies were the same as for immunofluorescence as described above. NSCs derived from E14 ES cells were utilised as a negative control for the detection of pluripotency and endodermal markers. FACS analysis was carried using the Attune Focusing Cytometer (Applied Biosystems, Thermo Fisher Scientific). The yellow-stained cells were excluded from the data.

### Statistical analysis

Statistical analysis was carried out using GraphPad Prism 7 software. Unless otherwise stated, the data show the mean and SEM from independent replicates, and statistical significance between parental and experimental lines was determined by one-way analysis of variance (ANOVA) and Dunnett’s multiple comparison test. Adjusted *p* values are indicated in the figure legends.

### Chromatin immunoprecipitation (ChIP)

Adherent cells were cross-linked in DMEM/F12 without serum for 5 min with 0.5% formaldehyde. ChIP was performed as described in [20]. Briefly, for each chromatin immunoprecipitation (IP) reaction, 5 µg of chromatin and 50 µl of magnetic DYNA beads–Protein A (10002D Thermo Fisher Scientific) were used. Magnetic beads were blocked with 0.5% BSA prior to incubating with 2–3 µl of antibody in 200 µl of blocking buffer. Antibodies were from Millipore: H3K4me3 (07-433), H3K27me3 (07-449), H3 CT pan (07-690), H3K27ac (07-360), H3K4me1 (07-436) or Abcam: H3122ac (ab33309). qPCR reactions contained 2% of IP DNA or 0.5 ng of input DNA. Primers were designed to target the H3K4me3 peaks identified from the publically available mouse ES cell data. Primer sequences and locations relative to each transcription start site (TSS) are listed in Additional file 2. TSSs for the indicated genes were confirmed by comparison with hCAGE data from the FANTOM5 project [47]. PCR conditions were as described for qRT-PCR above.

For ChIP-seq, libraries were prepared with the NEB ultra DNA library prep kit and single-end sequencing to 76-bp read length was carried out using an Illumina Nextseq machine by the University of Glasgow Polyomics facility. ChIPs from undifferentiated cells were performed and sequenced in triplicate, whereas ChIPs from differentiated cells were from a single replicate. Between 36 and 52 million reads were obtained from each sample. Quality was checked using FastQC, and the median Phred score per base over all reads for each sample was greater than 30. Unfiltered reads from FASTQ files were aligned to reference genome NCBI build 37 mm9 using Bowtie [48]; reads aligning to more than three positions and duplicate reads were discarded. Aligned reads were 64–75% of the total. Non-immune IgG was used as a negative control, and yielded 29 M reads with 55% aligning. Statistically enriched regions compared to a background control (peak finding) were identified using MACS v1.4 [49] with parameters *p* = 1e−3; bandwidth = 200; genome = mm9; fold enrichment = 10.30; and input control was used as a background. Putative enhancers were identified by overlapping peaks of H3K4me1 and H3K27ac from mouse ES Bruce4 cells (downloaded from the UCSC genome browser, accessions wgEncodeEM001682 and wgEncodeEM002709) [50, 51].

## Supplementary information

**Additional file 1.** Additional figures.

**Additional file 2.** Antibody dilutions, primer sequences and working concentrations.

## Data Availability

The ChIP-seq datasets supporting the conclusions of this article are available in the NCBI GEO repository under Accession Number GSE118031.
