# Supplementary material for: Maintenance of active chromatin states by HMGN2 is required for stem cell identity in a pluripotent stem cell model
Source: Epigenetics Chromatin. 2019 Dec 12;12:73. doi: 10.1186/s13072-019-0320-7 (PMC6907237; doi:10.1186/s13072-019-0320-7)
Supplement: Supplementary file 1 — Additional file 1. Additional figures. [file 13072_2019_320_MOESM1_ESM.pdf]

Additional file 1

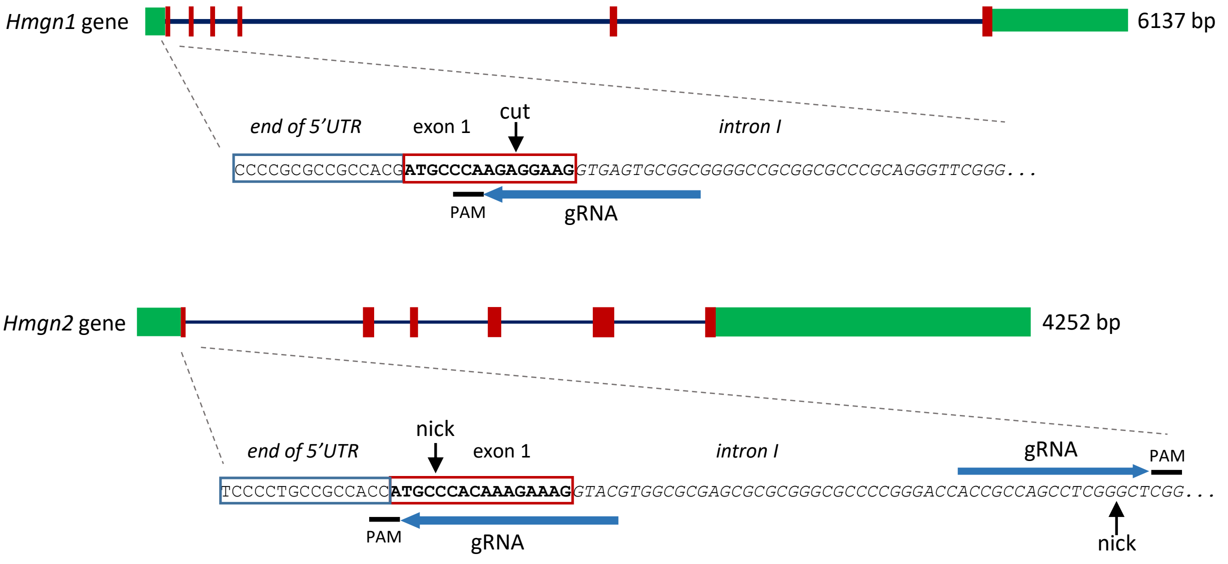

Figure S1: CRISPR mutagenesis strategies targeting the mouse *Hmgn1* and *Hmgn2* gene loci. In order to create a functional knockout of *Hmgn1*, a single guide was used to target wtCas9 to exon 1 of *Hmgn1* gene, and a different guide was used to concomitantly target the *Hprt* gene. The anticipated Cas9 cleavage site is 7 bp downstream of the *Hmgn1* ATG translation initiation codon, so insertion or deletion of base pairs during the DNA repair process could lead to a frameshift and loss of Hmgn1 protein production. For *Hmgn2*, a pair of guides was used to target the highly specific D10A Cas9 nickase mutant to exon 1 and the first intron. DNA sequencing revealed deletions of between 30 and 62 bp in both alleles of each *Hmgn2* knockout line (data not shown). The deletions cause frameshift mutations starting immediately before (N2-c) or after (N2-a, N2-b) the first ATG codon, and remove the intron I splice donor sequence, and result in complete loss of protein production.

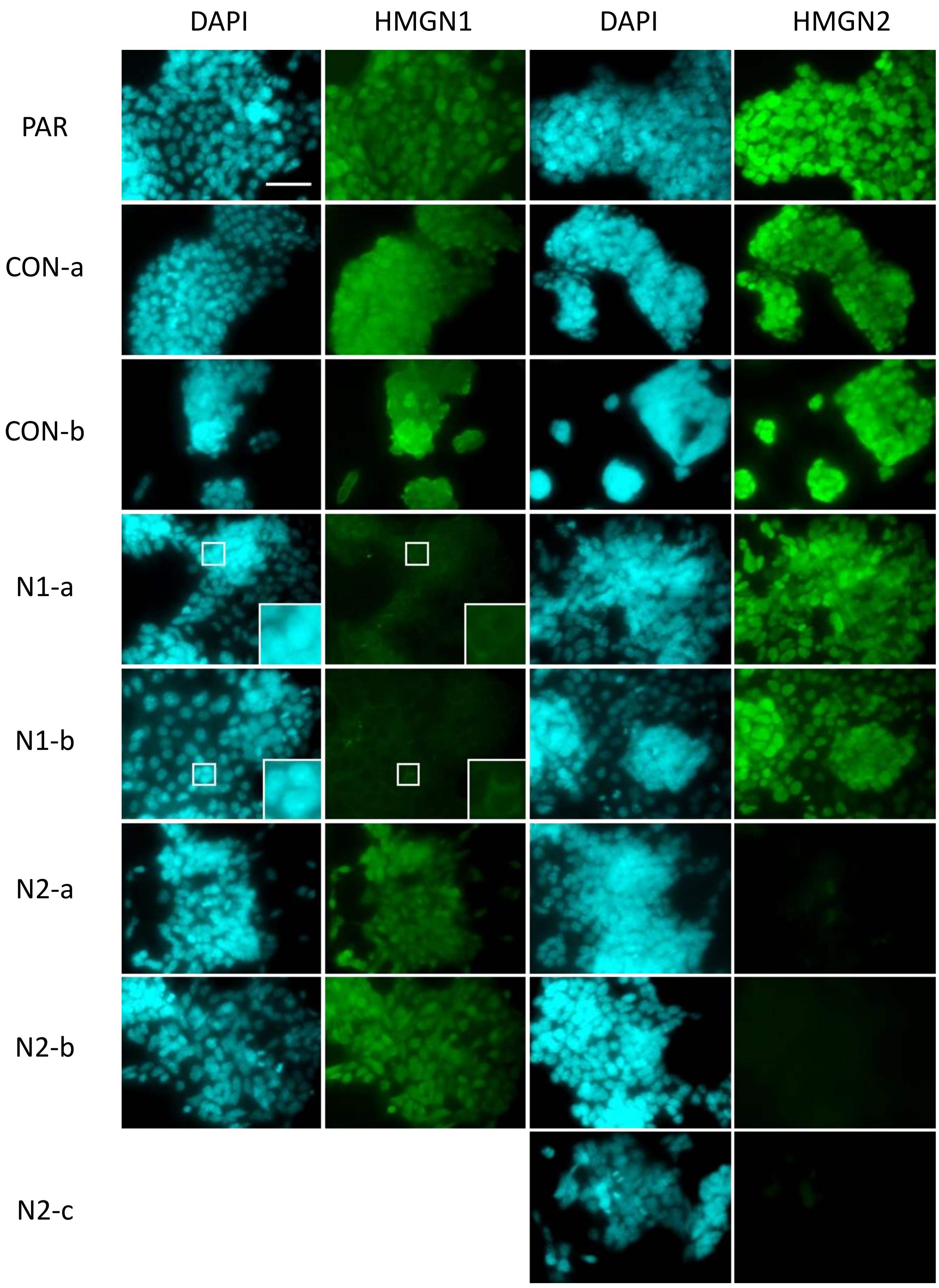

Figure S2: *Hmgn1-* and *Hmgn2*-targeted P19 cells lack nuclear nuclear staining for HMGN1 or HMGN2.

Immunofluorescence for the detection of HMGN1 or HMGN2 proteins (green). DAPI was used to stain the nuclei (cyan). Parental, CON-a, and CON-b cells contain both chromatin binding proteins. N2-a, N2-b, and N2-c cells lack HMGN2, but are positive for HMGN1, whereas N1-a and N1-b cells retain HMGN2. The 3X amplifications of the indicated cells highlight the loss of the nuclear signal corresponding to HMGN1 protein in N1-a and N1-b cells. Scale bar indicates 50 µm.

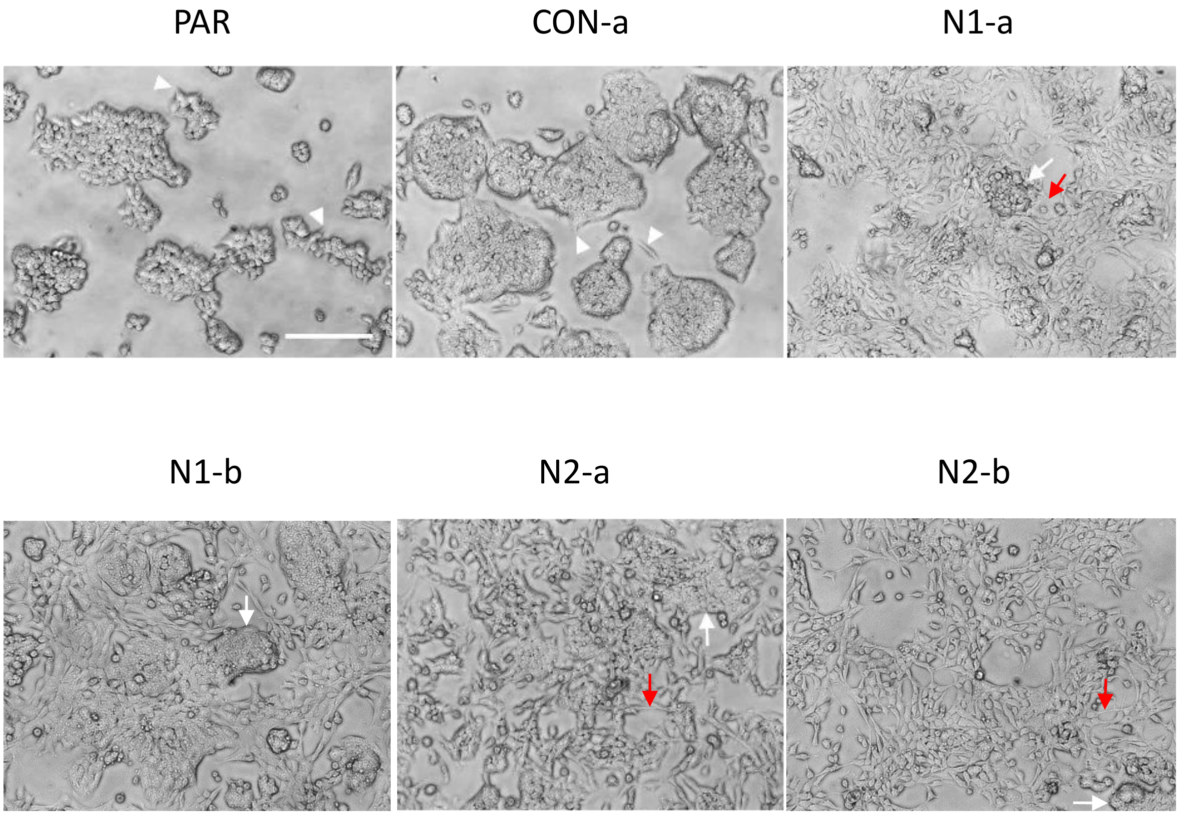

Figure S3: Colony morphology reveals substantial differences in cellular shape and organisation between parental and *Hmgn*-knockout cells

Clear field pictures of parental and *Hmgn*-knockout cultures that were plated at the same cell density. Parental and CON-a cells are morphologically homogeneous and form colonies, where the cells at the edges show minor cytoplasmic protuberances (arrow heads). Fewer distinct, compact colonies can be observed in the *Hmgn*-knockout cultures (white arrows) as most cells grow spread outside the colonies. The morphology of *Hmgn*-knockout cells is heterogeneous, and some cells have extended processes (red arrows) Scale bar indicates 200 µm.

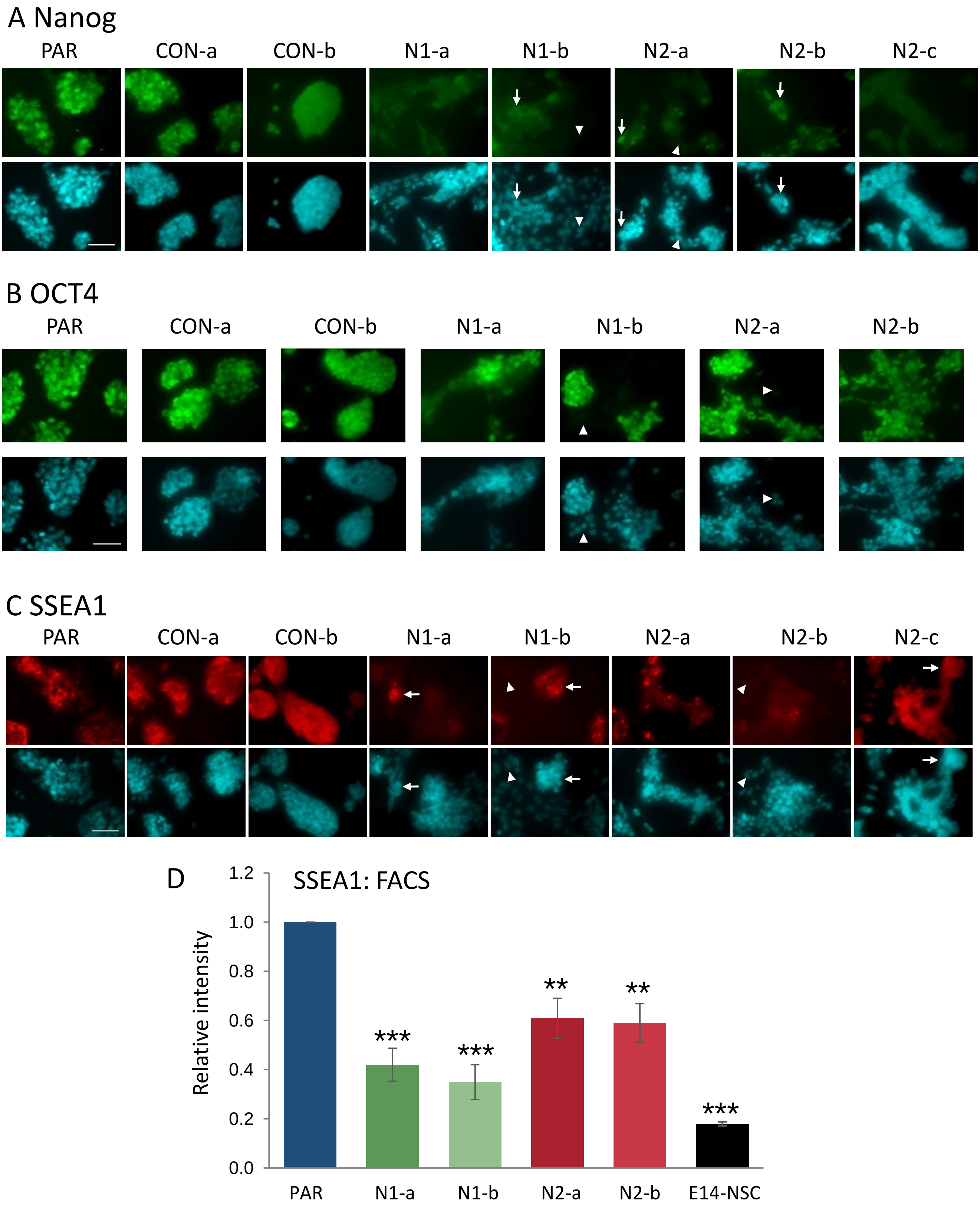

Figure S4: Immunostaining analysis of pluripotency marker gene expression

(A) Nanog immunofluorescence. Nanog levels are generally reduced in the *Hmgn* knockout lines, but there is some variation between cells, as shown by high (arrows) and reduced (arrow heads) fluorescence intensity. (B) OCT4 immunofluorescence. Arrowheads indicate cells with reduced OCT4 staining. (C) SSEA1 immunofluorescence. Clustered *Hmgn*-knockout cells retain higher levels of SSEA1 (arrows), whereas cells that grow outside the colonies tend to have lower expression (arrow heads). DAPI was used to stain the nuclei (cyan). Scale bar indicates 50 µm.

(D) FACS analysis of SSEA1 expression. The graph represents the relative fluorescence intensity (median) of each cell line, error bars symbolise the SEM from 3 independent experiments, and the statistical significance was calculated by ANOVA and Dunnett's multiple comparison test (adjusted p values *<0.05, **<0.001, ***<0.0001). E14-NSCs are a negative control.

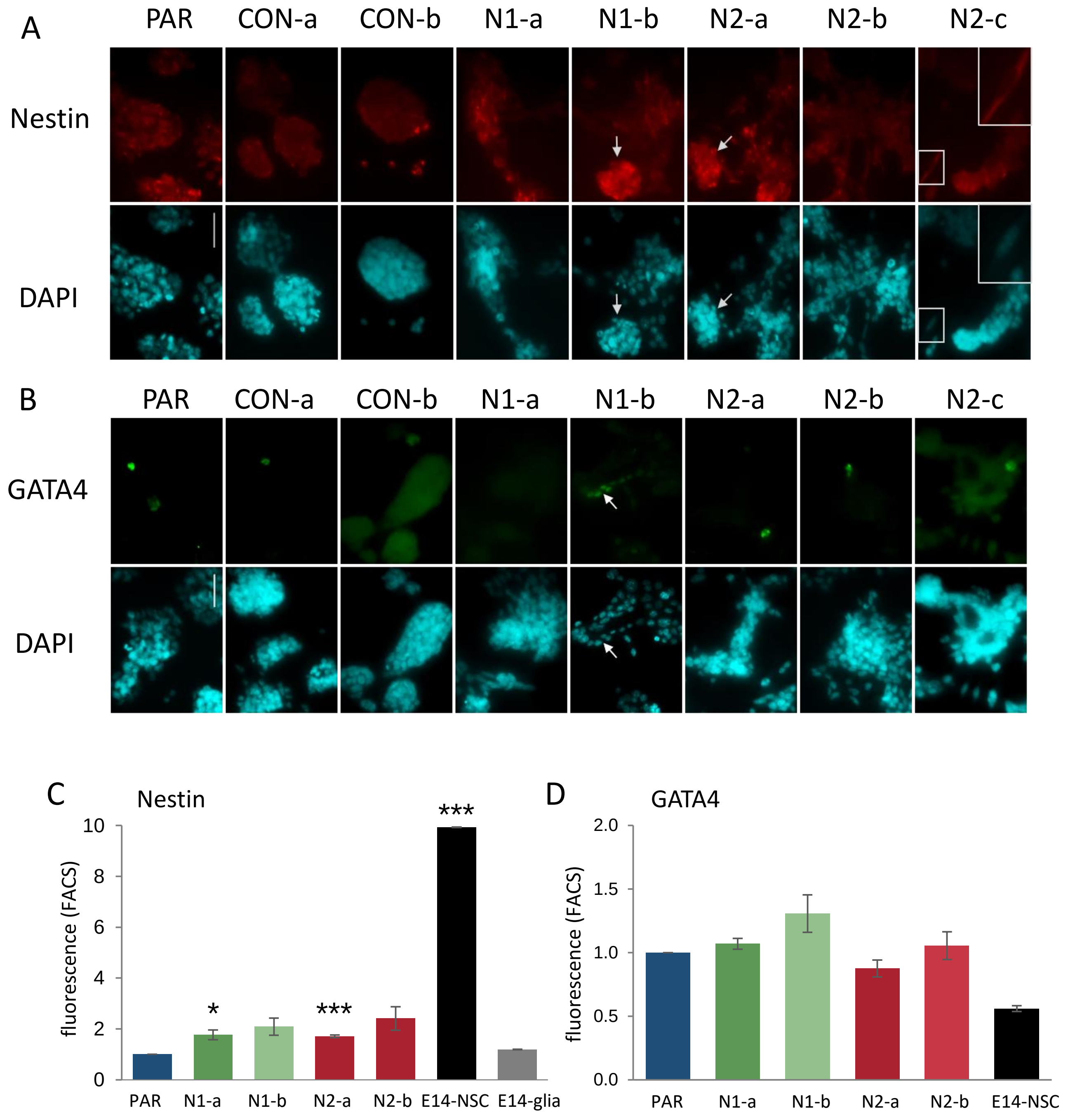

Figure S5: Nestin and GATA4 immunostaining in parental and *Hmgn* knockout cells
(A) Immunofluorescence for the intermediate filament Nestin (red). Fluorescence intensity corresponding to Nestin is higher in *Hmgn*-knockout cultures, especially in the three dimensional clusters (arrows). 2X amplifications (inset) reveal cytoskeleton projections in *Hmgn*-knockout cells. (B) Immunofluorescence for the endodermal transcription factor GATA4 (green). GATA4 is observed at similar levels in all the cultured cell lines, except in N1-b cultures which contain more GATA4-positive cells (arrows). DAPI was used to stain the nuclei (cyan). Scale bar indicates 50 µm.

C and D: FACS analysis of Nestin (C) and GATA4 (D) expression. Each graph represents the relative fluorescence intensity (median) of each cell line, error bars symbolise the SEM from 3 independent experiments, and the statistical significance was calculated by ANOVA and Dunnett's multiple comparison test (adjusted p values *<0.05, **<0.001, ***<0.0001). For Nestin staining, NSCs derived from E14 cells are a positive control, and E14-NSCs differentiated into glia cells are a negative control. For GATA4 staining, E14-NSCs are a negative control.

Spontaneous neuronal differentiation of *Hmgn2-knockout* cells is not driven by increased autocrine signalling

Three major extrinsic pathways that regulate neuronal differentiation are Notch signalling, which promotes survival and self-renewal of NS cells (Louvi et al, 2006), FGF signalling, which is important for both NS cell proliferation and differentiation (Wang et al, 2006), and WNT signalling, which can either promote self-renewal or neuronal specification, depending on the developmental stage (Hirabayashi et al, 2004). We hypothesised that these pathways may be dysregulated in the *Hmgn* knockout cells, leading to the increased levels of spontaneous neuronal differentiation that were observed.

Three known targets of these pathways were studied, *Axin2* (WNT pathway), *Fgf4* (FGF pathway) and *Hes5* (Notch pathway) (Figure S6a). No significant changes in expression of these target genes are observed in the *Hmgn2* knockout cell lines, and inhibition of Notch, FGF or WNT signalling reduces their expression as expected (Figure S6a), indicating that the activity of these signalling pathways is not altered in knockout cells.

Although Notch, FGF and WNT signalling does not appear to be increased in *Hmgn2* knockout cells, it is possible that the knockout cells respond differently to the signalling events that are active. Therefore, we investigated the effects of inhibiting these signalling pathways on the expression of the pluripotency gene *Nanog* and the pro-neural factor *Neurog1*. The response of these genes to the signalling inhibitors is the same in control and knockout lines (Figure S6b). Finally, immunofluorescence revealed that the proportion of neuronal cells is not altered following inhibition of WNT signalling, either in control or *Hmgn2* knockout cells (Figure S6c). Taken together, this data indicates that the autocrine WNT, FGF and Notch signalling pathways are not enhanced in *Hmgn2* knockout cells, and that the increased spontaneous neuronal differentiation does not appear to be driven by these signalling pathways.

Louvi, A. and Artavanis-Tsakonas, S. (2006) Notch signalling in vertebrate neural development. *Nat Rev Neurosci*, **7**, 93-102.

Wang, C., Xia, C., Bian, W., Liu, L., Lin, W., Chen, Y.G., Ang, S.L. and Jing, N. (2006) Cell aggregation-induced FGF8 elevation is essential for P19 cell neural differentiation. *Mol Biol Cell*, **17**, 3075-3084.

Hirabayashi, Y., Itoh, Y., Tabata, H., Nakajima, K., Akiyama, T., Masuyama, N. and Gotoh, Y. (2004) The Wnt/beta-catenin pathway directs neuronal differentiation of cortical neural precursor cells. *Development*, **131**, 2791-2801.

Figure S6 (*overleaf*): Inhibition of WNT, Notch or FGF signalling pathways does not alter the *Hmgn2* knockout cell phenotype (A) Relative expression of the reporter genes for WNT (*Axin2*), Notch (*Hes5*) and FGF (*Fgf-4*) signalling pathway activities and (B) relative expression of *Nanog* and *Neurog1* in parental, CON-a, and *Hmgn2*-KO cells, as determined by real time RT-PCR. Cells were seeded at a density of 2 x 10^5 cells/well in 6 well plates, allowed to attach overnight, then 10 μM of the signalling pathway inhibitor was added: DAPT for Notch, SU-5401 for FGFR, and XAV-939 for WNT. Expression was assayed after 24 h treatment. Data shows the mean and s.d. of 3 independent replicates. In (A), expression is plotted relative to that in untreated parental cells. Student's T-test was used to test for differences between untreated lines. * = $P < 0.05$. For each cell line in (B), expression in treated cells is plotted relative to that in untreated cells. The Student's T-test was used to test for significant differences between untreated and treated cells of the same line. * = $P < 0.05$, ** = $P < 0.01$. (C) βIII-tubulin immunostaining in PAR, N2-b and N2-c lines plated and grown in the presence or absence of WNT inhibitor (XAV-939) for 48 hrs.

Figure S6

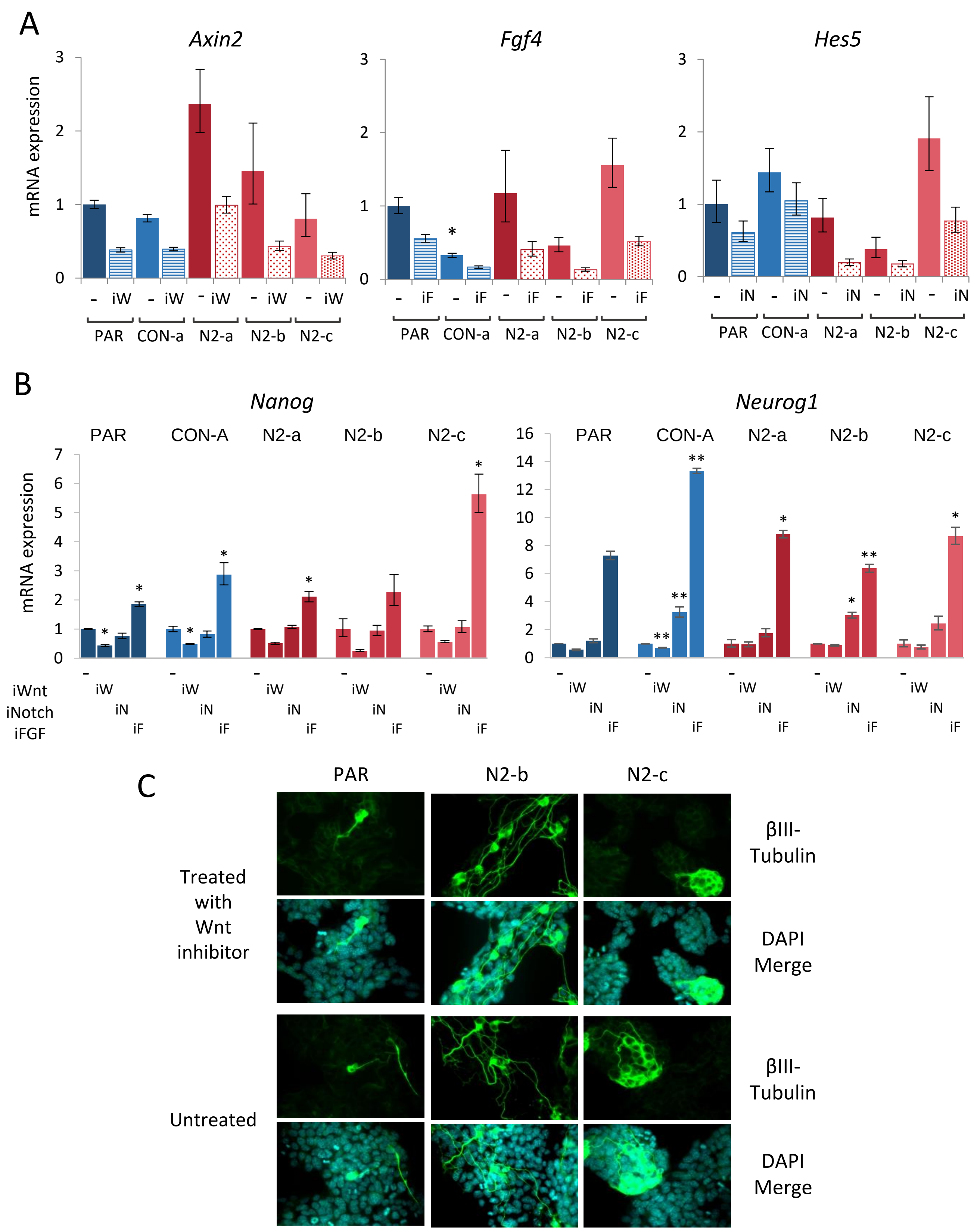
A
Axin2
Fgf4
Hes5
mRNA expression
3
2
1
0
- iW - iW - iW - iW - iW
PAR CON-a N2-a N2-b N2-c
- iF - iF - iF - iF - iF
- iN - iN - iN - iN - iN
*
B
Nanog
Neurog1
PAR CON-A N2-a N2-b N2-c
7
6
5
4
3
2
1
0
16
14
12
10
8
6
4
2
0
*
**
iWnt
iNotch
iFGF
- iW iN iF
C
PAR N2-b N2-c
Treated with Wnt inhibitor
Untreated
βIII-Tubulin
DAPI Merge

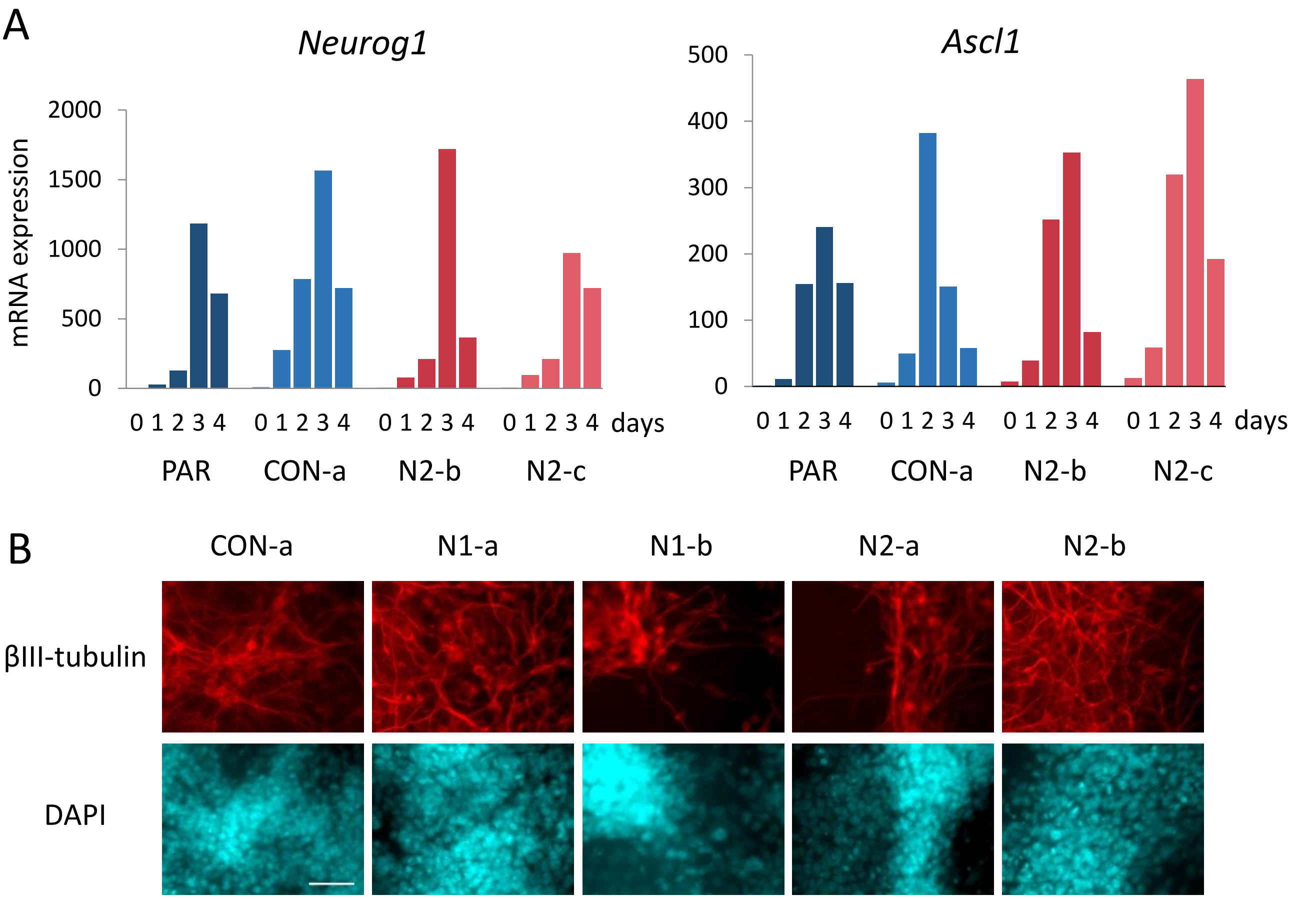

Figure S7: Induced neuronal differentiation is not altered in *Hmgn2* knockout cells.
(A) Relative mRNA expression of the *Neurog1* and *Ascl1* genes in parental, CON-a, and N2-b and N2-c cells induced to differentiate along the neuronal lineage. A total of four time points during the protocol were evaluated (1-4 days after induction), together with the undifferentiated cultures (day 0). Expression is plotted relative to that in undifferentiated, untreated parental P19 cells. The data is representative of two independent biological replicates.
(B) Immunofluorescence for βIII tubulin and DAPI at day 4 in parental, N1-a, N1-b, N2-a and N2-b cells. Scale bar indicates 50 μm.

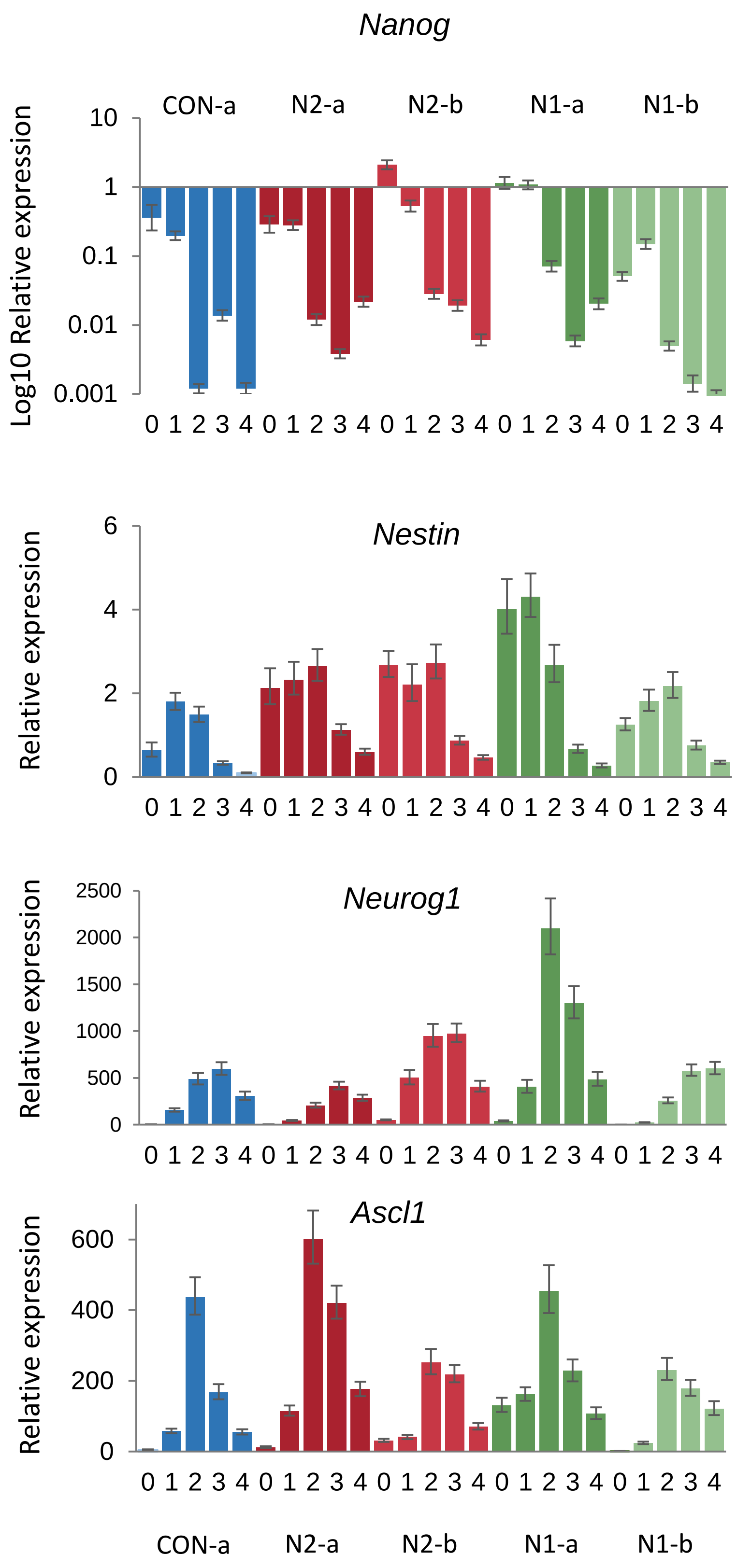

Figure S8: Neuronal induction of parental, CON-a, *Hmgn1*- and *Hmgn2*-knockout cells.
Relative expression of the indicated genes was determined by qRT-PCR in control, *Hmgn2* and *Hmgn1* knockout cells induced to differentiate along the neuronal lineage. A total of four time points during the protocol were evaluated (1-4 days), together with the undifferentiated cultures (day 0) in order to get the basal expression levels before differentiation. Expression was normalised to the endogenous housekeeping gene *Gpi1*. The normalised values were then compared with parental values at day 0. The graphs present the mean and standard deviation of technical triplicates from one biological replicate.

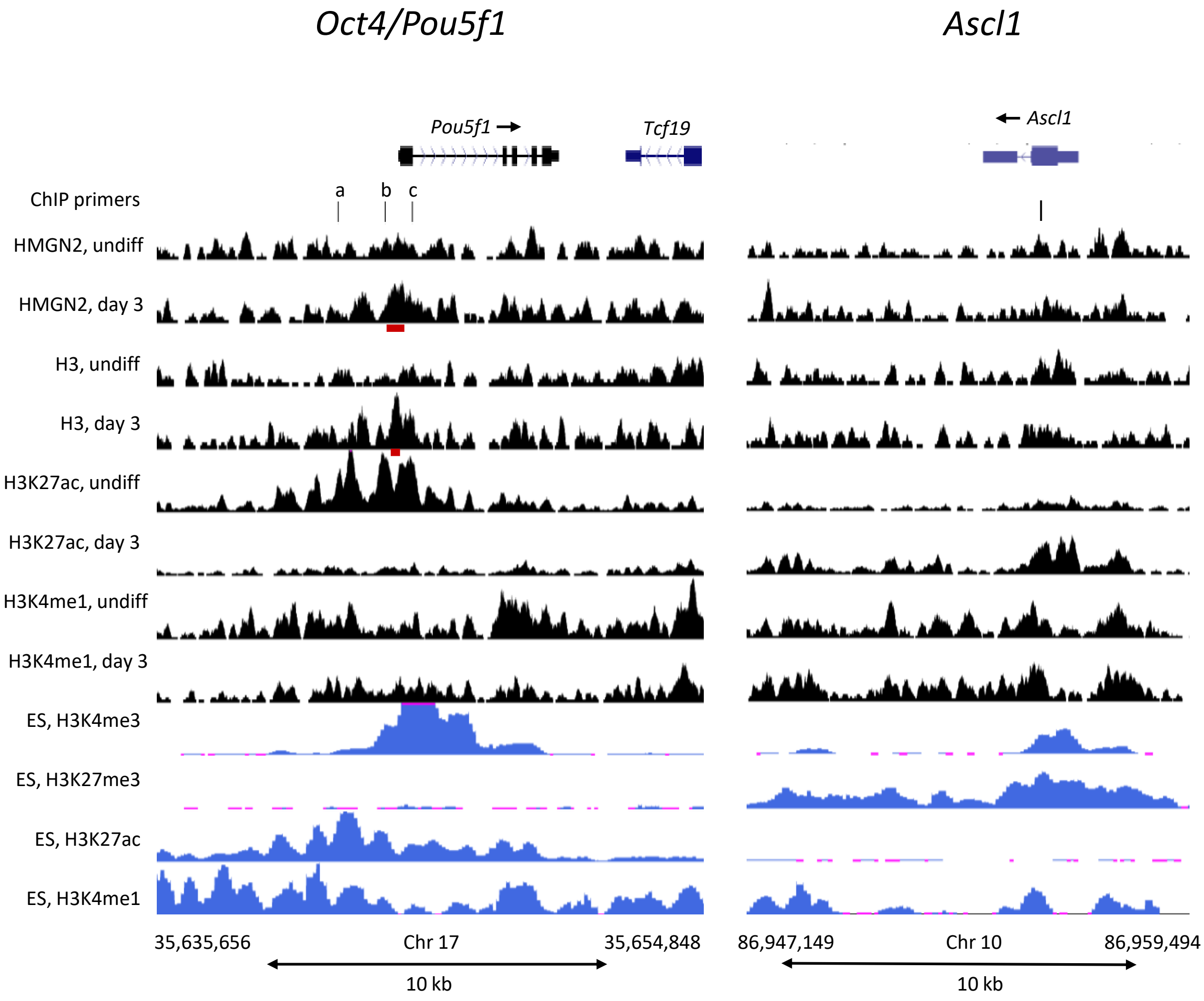

Figure S9: HMGN2 binding at the *Oct4* and *Ascl1* loci in P19 cells.
ChIP-seq for HMGN2, H3, H3K27ac and H3K4me1 was performed in undifferentiated and day 3 neuronal induced P19 cells. Reads were aligned to the mm9 mouse genome, and regions surrounding the *Oct4* (*Pou5f1*) and *Ascl1* loci are shown. Peaks revealed by MACS peak calling software for HMGN2 and H3 are shown as blocks below the relevant signal track. Data for H3K4me3, H3K27me3, H3K27ac and H3K4me1 in mouse ES cells was obtained from the UCSC genome browser; accession numbers are wgEncodeEM001682, wgEncodeEM002709, wgEncodeEM002497 and wgEncodeEM001681. Y-axis scales are: 0 – 0.62 for all H3 and HMGN2 tracks; 0 – 1.7 for H3K27ac; 0 – 0.9 for H3K4me1, 0 – 33.4 for H3K4me3 in ES cells at *Oct4* and 0 – 9.8 at *Ascl1*.

A

		TSS	enhancers
	peaks	37681	13025
HMGN2	1862	26	126

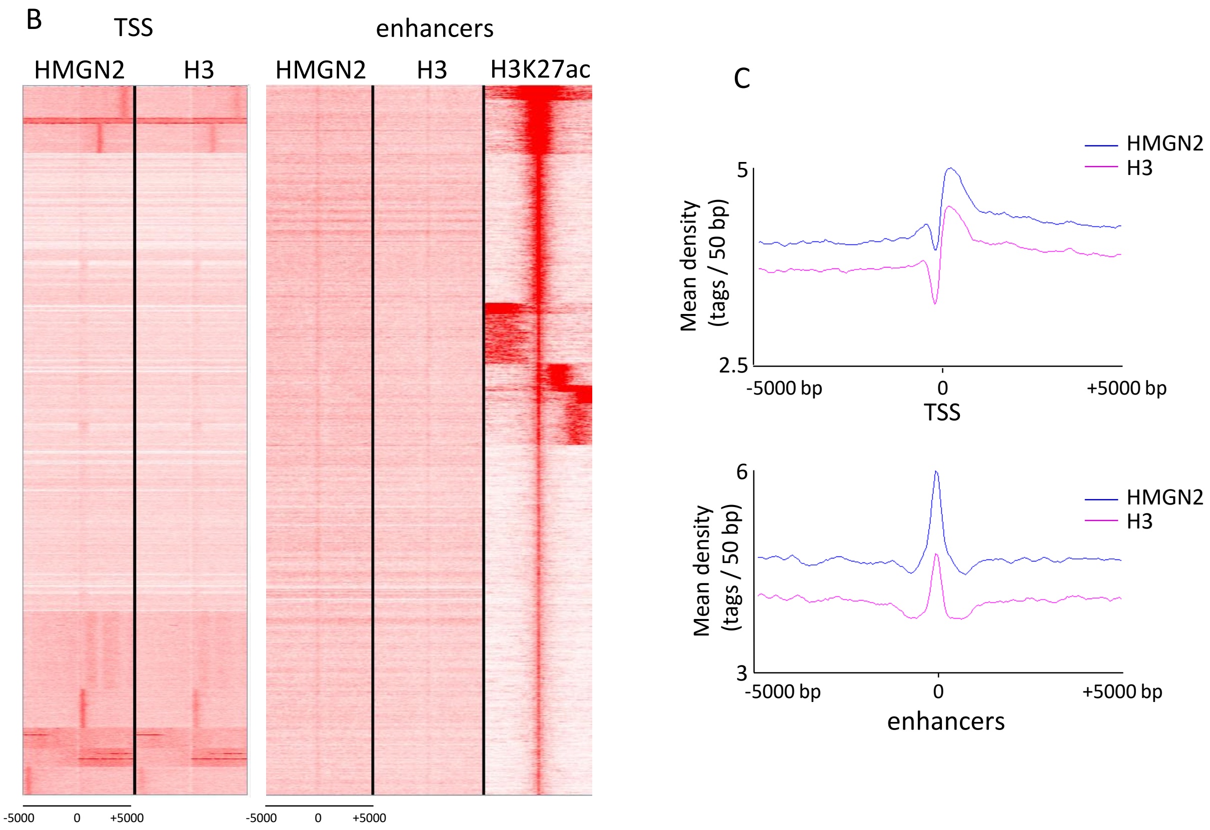

Figure S10: HMGN2 is not highly enriched at transcription start sites and enhancers
(A) The number of HMGN2 peaks that overlap with transcription start sites (TSS) or putative enhancers. (B) KMeans clustering of tag density arrays showing the density of HMGN2 and H3 ChIP-seq reads for 5000 bp upstream and downstream of RefSeq TSSs or putative enhancers. H3K27ac reads from Bruce4 ES cells is included as a control for the enhancers analysis. This mark is typically found at enhancers and active promoters. Data was analysed using SeqMINER (Ye et al, 2011) (C) Plots of the mean tag density for HMGN2 and H3 around TSSs and enhancers. For both TSSs and enhancers, the density of HMGN2 has a similar profile to that of H3, providing no evidence that HMGN2 is preferentially enriched at either type of element.

Ye T, Krebs AR, Choukrallah MA, et al. SeqMINER: an integrated ChIP-seq data interpretation platform. *Nucleic Acids Res*. 2011;39(6)

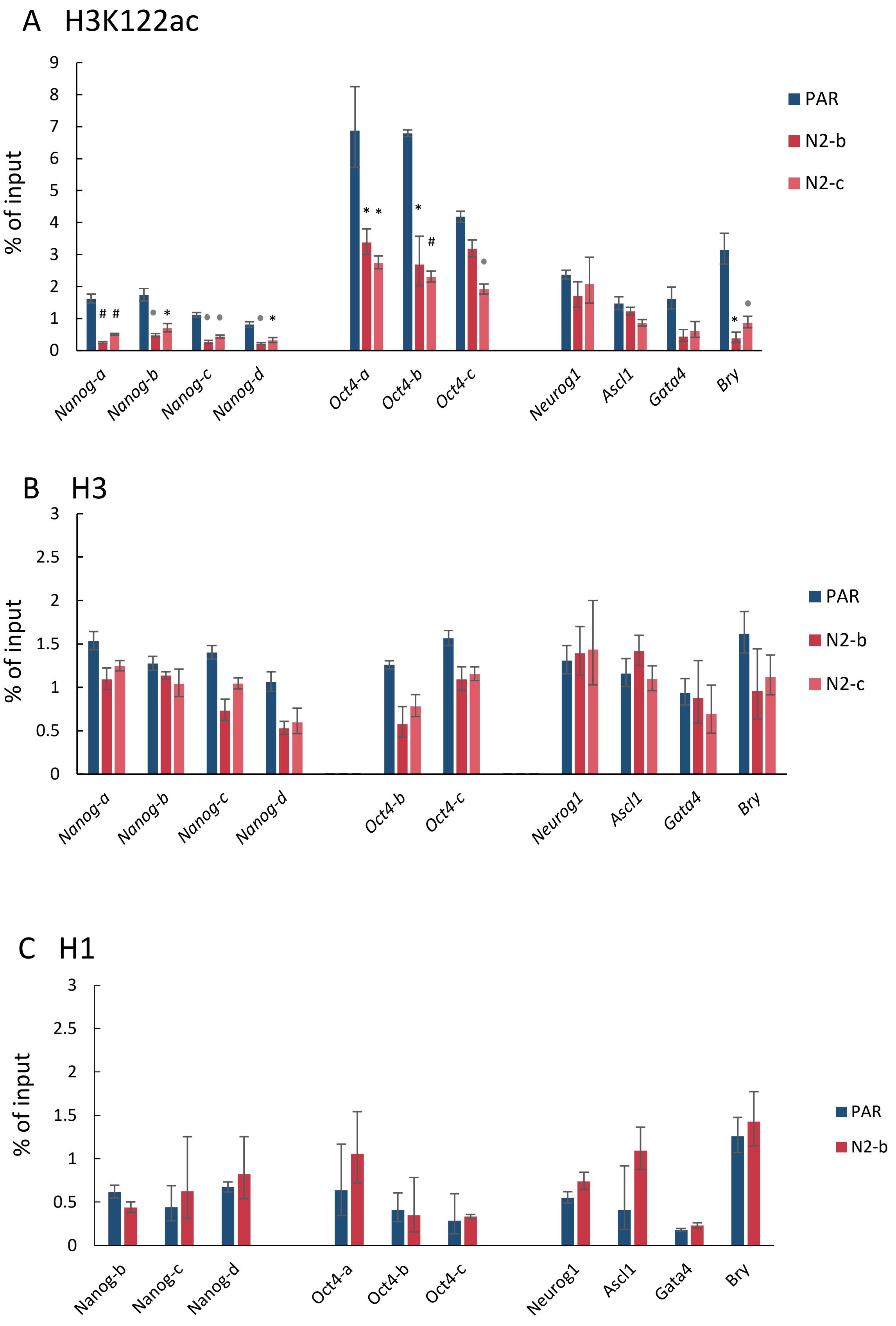

Figure S11: Chromatin immunoprecipitation analysis in *Hmgn2* knockout cells.
ChIP-PCR assays for H3K122ac (A) , H3 (B) and H1 (C). Data shows the enrichment of each ChIP as a percentage of input. Statistical significance between parental and experimental lines was determined using the Student's T test, with Bonferroni correction for multiple testing (* p < 0.005, • p < 0.001, # p < 0.0001).
