# Supplementary material for: Maintenance of active chromatin states by HMGN2 is required for stem cell identity in a pluripotent stem cell model
Source: Epigenetics Chromatin. 2019 Dec 12;12:73. doi: 10.1186/s13072-019-0320-7 (PMC6907237; doi:10.1186/s13072-019-0320-7)
Supplement: Supplementary file 2 — Additional file 2. Antibody dilutions, primer sequences and working concentrations. [file 13072_2019_320_MOESM2_ESM.pdf]

Additional file 2

Antibody dilutions used

Antibody	Provider	Catalogue number	IF/FACS dilution	WB dilution	ChIP vol/rxn
Rb anti POU5F1	Abcam	Ab19857	1:500		
Rb anti NANOG	Abcam	Ab80892	1:200		
Ms anti SSEA1	Abcam	Ab16285	1:200		
Rb anti GATA4	Abcam	Ab84593	1:500		
Ms anti NES	Abcam	Ab6142	1:200		
Rb anti TUBB3	Abcam	Ab18207	1:2000		
Ms anti TUBB3	Millipore	MAB1637	1:500		
Ch anti MAP2	Abcam	Ab5392	1:2000		
Rb anti GFAP	Abcam	Ab7260	1:500		
Rb anti HMGN1	Custom (Eurogentec)		1:1000	1:1000	5 µl
Rb anti HMGN2	Custom (Eurogentec)		1:2000	1:1000	5 µl
Rb anti H3	Millipore	07-690		1:25000	2 µl
Rb anti H3K4me3	Millipore	07-473		1:20000	5 µl
Rb anti H3K27me3	Millipore	07-449			7.5 µl
Rb anti H3K9ac	Millipore	07-352		1:10000	5 µl
Rb anti H3K27ac	Millipore	07-360		1:20000	5 µl
Rb anti H3K122ac	Abcam	Ab33309			5 µl
Ms anti TUBB	TFS	MA5-16308		1:3000	
rIgG	Sigma	I5006			7.5 µl
mIgG	Sigma	12-371	1:200		
Gt anti rb-HRP	TFS	32460		1:1000	
Dn anti rb-488	TFS	A21206	1:1000		
Dn anti rb-594	TFS	A11037	1:1000		
Gt anti ms-594	TFS	A11020	1:1000		
Gt anti ch-594	TFS	A11042	1:1000		

gRNA protospacer sequences

MmHN2ATG A69 5’-GTACCTTTCTTTTGGGCA
MmHN2ATG B77 5’-ACCGCCAGCCTCGGGCT
mN1ex1g3 5'-GCCGCACTCACCTTCCTCT
MmHPRT (exon 7) 5'-GGGGCTGTACTGCTTAACCA

qRT-PCR primer sequences (final concentration in PCR reaction indicated after each sequence)

Hmgn1 F AGAGACGGAAAACCAGAGTCCAG 300 nM
R CGTGATGGATGCTTAGTCGGA 300 nM

Hmgn2 F AAAAGGCCCCTGCGAAGAA 300 nM
R TGCCTGGTCTGTTTTGGCA 300 nM

Nestin F AAAGTTCCAGCTGGCTGT 300 nM
R CACTTCCAGACTAAGGGACAT 300 nM

Map2 F TCTGCCTCTAGCAGCCGAAG 300 nM
RCACTGTGGCTGTTTGTTCTG 300 nM

Oct4F CGTTCTCTTTGGAAAGGTGTTCA 600 nM
R GGTTCTCATTGTTGTCGGCTTC 600 nM

Nanog F ACCTGAGCTATAAGCAGGTTAAG 600 nM
R TCAGACCATTGCTAGTCTTC 600 nM

Sox2 F CGTTCATGTGCGCGTAGCTG 600 nM
R GGAACAGCATGGCGAGCGG 600 nM

Gpi1 F TCCGTGTCCCTTCTCACCAT 900 nM
R TGGCAGTTCCAGACCAGCTT 50 nM

Neurog1 F GGCTTCATGCATTATGGATCC 900 nM
R CTCCAGTCCAGTGCCTGAATAG 900 nM

Ascl1 F CCAACTGGTTCTGAGGACCTG 600 nM
R CTGCCATCCTGCTTCCAAA 600 nM

Gata4 F TAGTCTGGCAGTTGGCACAG 300 nM
R ACGGGACACTACCTGTGCAA 300 nM

Bry (Tbxt) F ATGCCAAAGAAAGAAACGAC 300 nM
R AGAGGCTGTAGAACATGATT 300 nM

bIII tubulin F AAGGTAGCCGTGTGTGACATC 300 nM
R ACCAGGTCATTCATGTTGCTC 300 nM

Axin2 F GAGAGTGAGCGGCAGAGC 300 nM
R CGGCTGACTCGTTCTCCT 300 nM

Fgf4 F CGACCACAGGGACGCTGCTG 300 nM

R ACTCCGAAGATGCTCACCACG 300 nM

Hes5 F CACCAGCCCAACTCCAAGCT 300 nM
R GGCGAAGGCTTTGCTGTGT 300 nM

Gfap F CAACCTGGCTGCGTATACCAG 600 nM
R TTAAGAACTGGATCTCCTCC 600 nM

ChIP primer sequences

Gata4-169_F CCAACAGGCAAAGTCCATGC 300 nM
Gata_R CACTGAGGGCAGAACGGAG 300 nM
Bry+11_F TGTAATCTTTGGGCTCCGCA 600 nM
Bry_R CCTACCCAACAGCCACCTTC 600 nM
Ascl1+1014_F CGTCTCCACCTTGCTCATCT 300 nM
Ascl1_R TTGGTCAACCTGGGTTTTGC 300 nM
Neurog1+295_ F GGTGAGGAAGCTGGACAGG 300 nM
Neurog1_R CCCTTTGGAGACCTGCATCT 300 nM
Nanog-1081_F (Nanog-a) GGAAGAACCACTCCTACCAATACTCA 300 nM
Nanog-1081_R (Nanog-a CGTAACATCTCCCATGTGAAGACTC 900 nM
Nanog-219_F (Nanog-b) TCTTTAGATCAGAGGATGCCCCCTAAGC 300 nM
Nanog-219_R (Nanog-b) AAGCCTCCTACCCTACCCACCCCCTAT 300 nM
Nanog+929_F (Nanog-c) TCAGCCCAGTACTCAGGCTTGT 300 nM
Nanog+929_R (Nanog-c) AGCCTAGCAGCCTCTTGGTTCT 300 nM
Nanog+1740_F (Nanog-d) TAACTGGACCCTCTGACTGGCT 300 nM
Nanog+1740_R (Nanog-d) CCCACCATCTTTTCTGCTAGTACAAG 300 nM
Oct-1781_F (Oct4-a) GTGAGCATGACAGAGTGGAGGAA 300 nM
Oct-1781_R (Oct4-a) TCTCTGGCCCTCTCCATGAAT 900 nM
Oct-399_F (Oct4-b) GTGGGTAAGCAAGAACTGAGGA 300 nM
Oct-399_R (Oct4-b) TGGAGAGCCTAAAACATCCATT 900 nM
Oct+410_F (Oct4-c) CAATGCCGTGAAGTTGGAGA 300 nM
Oct+410_R (Oct4-c) TCACTTACCTCCTCGGGAGTTG 900 nM
